# Strengthening D-A Push–Pull Interactions in BODIPY to Enhance Near-Infrared Absorption and Photothermal Conversion for Low-Intensity Photothermal Antitumor Therapy

**DOI:** 10.3390/molecules31132258

**Published:** 2026-06-26

**Authors:** Yamin Li, Xiaolu Weng, Jianyong Liu

**Affiliations:** Key Laboratory of Molecule Synthesis and Function Discovery, Fujian Province University, College of Chemistry, Fuzhou University, Fuzhou 350108, China; 15236954674@163.com (Y.L.); 15213509135@163.com (X.W.)

**Keywords:** BODIPY, photothermal conversion efficiency, photothermal therapy, push-pull electronic effect, nanoparticles

## Abstract

Conventional photothermal therapy often relies on high-intensity laser excitation due to the limited photothermal conversion efficiency (PCE) of existing photothermal agents (PTAs), which compromises treatment safety and restricts clinical translation. To address this limitation, we designed and synthesized a series of boron-dipyrromethene (BODIPY)-based derivatives (BDP 1–4) featuring gradient-enhanced donor–acceptor (D-A) push–pull electronic effects for efficient photothermal antitumor therapy. The structure–activity relationships were systematically elucidated through photophysical characterization and in vitro/in vivo photobiological evaluation. From BDP 1 to BDP 4, the progressively strengthened push–pull effect leads to enhanced intramolecular charge transfer (ICT), which, in turn, results in a narrowed HOMO-LUMO gap, redshifted absorption into the near-infrared (NIR) region (up to 843 nm), markedly attenuated fluorescence emission, and a remarkable increase in PCE up to 88.3%. To improve water dispersibility and tumor targeting, these molecules were further encapsulated into nanoparticles using DSPE-PEG_2000_, and the nanoformulations retained high PCE. Both in vitro and in vivo studies demonstrated that under low-power laser irradiation (0.5 W·cm^−2^, 808 nm), the nanoformulation of BDP 4, which exhibited the highest PCE among the series, achieved pronounced photothermal tumor ablation without inducing systemic toxicity. Overall, this study proposes a molecular design strategy that synergistically modulates NIR absorption and photothermal conversion by enhancing the D-A push–pull effect. This strategy provides a design rationale for developing efficient, low-toxicity organic PTAs, and demonstrates potential applicability in low-power PTT modalities.

## 1. Introduction

Malignant tumors remain one of the most formidable public health challenges worldwide, with persistently high incidence and mortality rates posing a continuous and significant threat to human life and health [1]. According to the latest data from the International Agency for Research on Cancer (IARC), the global annual number of new cancer cases exceeds 20 million, with nearly 10 million deaths, and the disease burden continues to escalate [2]. Currently, conventional therapeutic modalities such as surgery, chemotherapy, and radiotherapy are widely used but each suffers from inherent limitations. Surgery is invasive and ineffective against metastatic lesions, and chemotherapy is associated with severe systemic toxicity and often induces drug resistance, while radiotherapy may damage surrounding normal tissues [3,4,5,6,7]. Therefore, the development of novel antitumor strategies that are highly effective, low toxic, and capable of precise targeting is urgent and highly needed.

Photothermal therapy (PTT), as an emerging non-invasive local treatment modality, offers a highly promising approach to addressing the above challenges [8,9]. PTT relies on photothermal agents (PTAs) with near-infrared (NIR) absorption to efficiently convert light energy into thermal energy under external laser irradiation, thereby generating localized hyperthermia (>50 °C) within the tumor and achieving selective ablation of cancer cells [10,11,12]. This technique demonstrates notable advantages, including high spatiotemporal selectivity, low systemic toxicity, controllable operation, and a reduced tendency to induce drug resistance [13].

Photothermal agents are the core components of PTT technology. In recent years, organic small-molecule photothermal agents have gradually emerged as the research frontier in this field, surpassing inorganic nanomaterials such as gold nanorods and copper sulfide, which face challenges including long-term accumulation, poor biodegradability, and potential metal-induced toxicity. This shift is attributed to the distinct advantages of organic agents, including well-defined chemical structures, tunable physicochemical properties, good biocompatibility, and clear metabolic pathways [14,15,16,17,18]. Among various organic dyes, boron-dipyrromethene (BODIPY) derivatives have demonstrated considerable potential for PTT owing to their high molar extinction coefficients, excellent photostability, and highly modifiable molecular scaffolds [19,20,21]. Through molecular engineering strategies such as extending the conjugated system, constructing D-A structures, or modulating molecular aggregation states (e.g., J-aggregation), the excited-state relaxation pathways of organic dyes can be effectively regulated, radiative transitions can be suppressed, and more energy can be channeled into non-radiative thermal vibration pathways, thereby achieving highly efficient photothermal conversion [22,23,24,25,26,27].

However, translating the excellent in vitro performance of BODIPY dyes into efficient in vivo therapy still faces two major bottlenecks. First, the absorption wavelength is limited. The maximum absorption of most BODIPY derivatives lies within the visible region (400–700 nm), which restricts tissue penetration depth and severely hampers therapeutic efficacy against deep-seated or visceral tumors [28,29]. However, biological tissues possess a “tissue transparency window” in the near-infrared (NIR) region (700–950 nm), within which light absorption and scattering are significantly reduced, and penetration depth is markedly increased. Second, overall performance remains insufficient. Both the photothermal conversion efficiency (PCE) and the tumor-targeting accumulation capability need further improvement, especially under low-dose administration and low-power irradiation, where achieving adequate therapeutic intensity and signal-to-noise ratio becomes challenging [30,31,32,33]. Therefore, designing and synthesizing BODIPY-based phototheranostic agents that absorb in the near-infrared “biological optical window” (NIR-I, 700–950 nm), possess high PCE, exhibit good targeting ability, and are suitable for low-dose treatment regimens represents a crucial step toward advancing their clinical translation.

**Figure 1:**
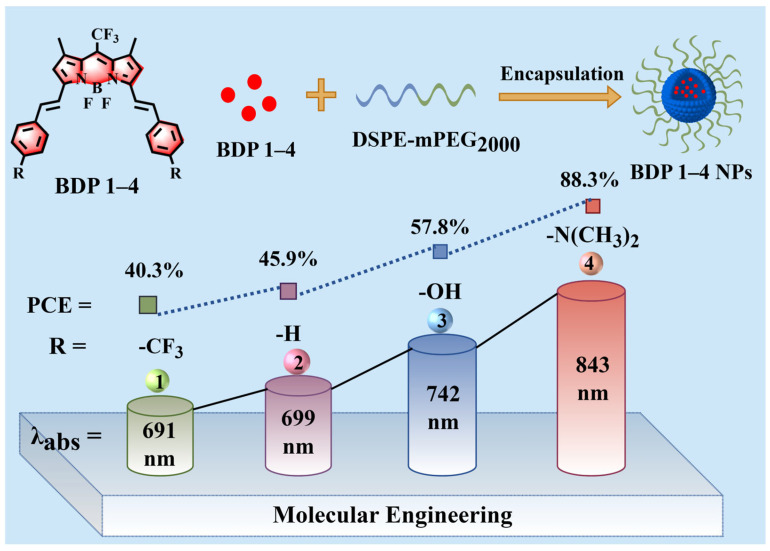
Molecular engineering and photophysical properties of BDP 1–4.

To this end, several BODIPY-based photothermal systems have been reported [34,35], yet a systematic study that progressively tunes the donor–acceptor (D-A) strength within a single BODIPY platform to investigate the stepwise structure–property relationships among absorption, fluorescence emission, and photothermal conversion efficiency is still lacking. Here, we hypothesize that gradually increasing the electron-donating ability of substituents on a strongly electron-withdrawing BODIPY core will stepwise enhance the intramolecular D-A effect, thereby promoting intramolecular charge transfer, narrowing the HOMO-LUMO gap, and causing a stepwise redshift in absorption, which, together with fluorescence quenching, should synergistically boost PCE. To test this hypothesis, this study proposes a systematic molecular design strategy: using a BODIPY core bearing a strongly electron-withdrawing meso-trifluoromethyl (-CF_3_) substituent, and sequentially introducing substituents with gradually increasing electron-donating ability (-CF_3_, -H, -OH, -N(CH_3_)_2_) at the para position of the 3,5-styryl groups, thereby obtaining a series of derivatives (BDP 1–4) that exhibit a gradient-enhanced D-A push–pull electronic effect (Figure 1). Based on this, the regulatory effect of the D-A push–pull effect on photophysical properties (absorption/fluorescence) and photothermal conversion efficiency was systematically investigated. The results show that as the D-A effect strengthens, the HOMO-LUMO energy gap gradually decreases, the absorption spectra redshift into the near-infrared region, the fluorescence emission progressively weakens, and the PCE increases accordingly, reaching up to 88.3%. To further enhance tumor targeting ability, these molecules were encapsulated into nanoparticles using DSPE-PEG_2000_. Through in vitro cell experiments and in vivo animal model validation, the optimal BDP 4 nanoformulation exhibits significant antitumor activity and good biosafety under relatively low-drug-concentration and low-laser-dose conditions. These findings reveal the molecular design principle of synergistically modulating the absorption wavelength and PCE by enhancing the D-A effect, and provide experimental basis and design reference for the development of high-performance near-infrared organic photothermal theranostic agents.

## 2. Results

### 2.1. Molecular Design and Chemical Synthesis

**Scheme 1:**
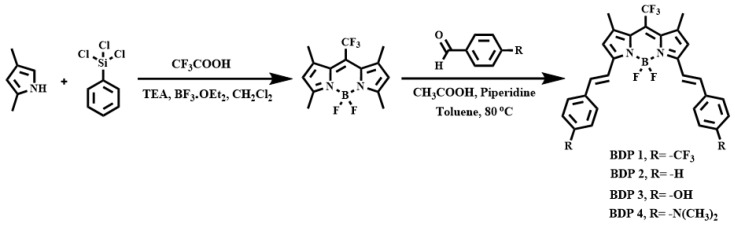
Synthesis of BDP 1–4.

The parent BODIPY core typically exhibits maximum absorption in the visible region, which severely limits its application in PTT due to shallow tissue penetration and strong absorption by endogenous chromophores such as hemoglobin. In contrast, near-infrared (NIR) light offers deeper tissue penetration, lower photon energy, and the ability to “bypass” normal tissues, thereby enabling precise focusing on tumor sites and efficient conversion into heat for selective therapy [36,37]. Based on this rationale, we designed a series of BODIPY derivatives (BDP 1–4) to redshift their absorption into the NIR region through two synergistic strategies: (1) π-conjugation extension by introducing styryl groups at the 3,5-positions of the BODIPY core to expand the conjugated system and narrow the energy gap; and (2) construction of a gradient-enhanced D-A push–pull system by incorporating a strong electron-withdrawing trifluoromethyl (-CF_3_) group at the meso position, while progressively strengthening the electron-donating ability of the para-substituents on the styryl rings. This D-A push–pull effect enhances ICT, thereby narrowing the HOMO-LUMO energy gap and shifting the absorption peak to the near-infrared (NIR) region, accompanied by a progressive attenuation of fluorescence emission and a corresponding increase in photothermal conversion efficiency. The specific synthetic route is illustrated in Scheme 1. First, the BDP parent compound substituted with a trifluoromethyl group was synthesized according to a literature procedure [38]. Next, this compound underwent Knoevenagel condensation with various para-substituted benzaldehydes bearing different electronic characteristics (para-substituents: -CF_3_, -H, -OH, and -N(CH_3_)_2_) to afford a series of conjugated-extended BODIPY derivatives BDP 1–4 with graded push–pull electronic strengths. To the best of our knowledge, although the tetramethyl-substituted BDP-CF_3_ precursor has been used in Knoevenagel condensation by others [29], those studies focused on single substituents or specific D-A pairs and did not systematically explore the stepwise structure–property relationships. In contrast, our work constructs a complete electron-donating gradient series (-CF_3_, -H, -OH, -N(CH_3_)_2_) on the same molecular platform, revealing, for the first time, a systematic relationship which provides a rational design strategy for high-performance photothermal BODIPY derivatives. The chemical structures of all the four compounds were fully confirmed by ^1^H NMR, ^13^C NMR, and high-resolution mass spectrometry (HRMS).

### 2.2. Optical Properties

**Figure 2:**
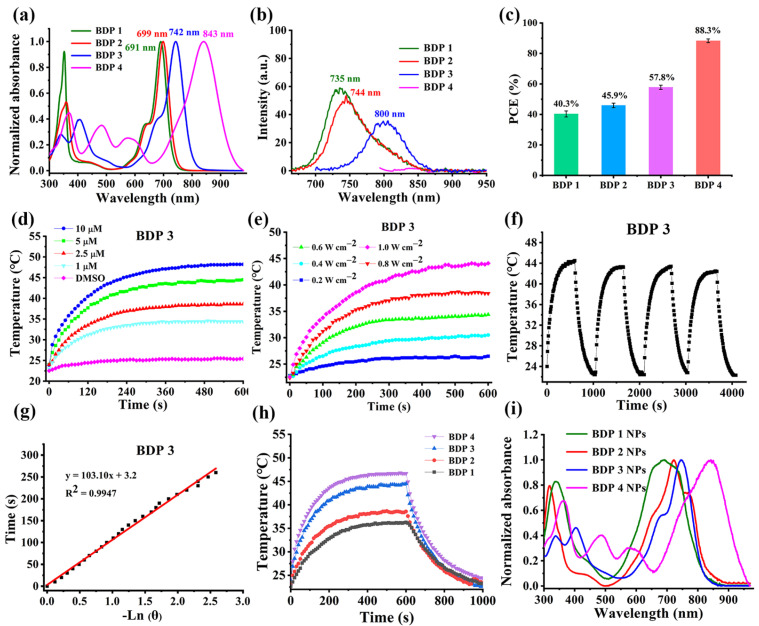
Optical properties. (**a**,**b**) Normalized electronic absorption (**a**) and fluorescence emission (**b**) spectra of BDP 1–4 in DMSO. (**c**) Comparison of PCE among BDP 1–4. (**d**) Temperature elevation curves of BDP 3 at different concentrations under 730 nm laser irradiation (0.8 W·cm^−2^). (**e**) Temperature elevation curves of BDP 3 (5 μM) under different laser power densities. (**f**) Temperature variation in BDP 3 over four heating/cooling cycles under 730 nm laser irradiation. (**g**) Linear relationship between −Ln(θ) and time for BDP 3. (**h**) Comparison of temperature variation profiles of BDP 1–4 under a laser irradiation and cooling cycle. (**i**) Normalized absorption spectra of BDP 1–4 NPs. Data are presented as mean ± SD (*n* = 3).

To elucidate the influence of the electronic properties of the para-substituents on the 3,5-styryl groups of the BODIPY core on its photophysical behavior, the UV–vis absorption spectra of BDP 1–4 in DMSO were first recorded. As shown in Figure 2a, the Q-band absorption maxima of the four compounds were located at 691, 699, 742, and 843 nm, respectively, exhibiting a distinct stepwise redshift as the intramolecular D-A electronic effect strengthened. Correspondingly, their fluorescence emission peaks also shifted bathochromically from BDP 1 to BDP 4, while the fluorescence intensity decreased significantly (Figure 2b). This spectral trend is primarily attributed to the progressively increasing electron-donating ability of the para-substituents: -CF_3_ < -H < -OH < -N(CH_3_)_2_. Together with the strong electron-withdrawing -CF_3_ group at the meso position, these molecules construct an intramolecular D-A push–pull system of gradually increasing strength. Previous investigations have demonstrated that enhanced D-A interaction promotes intramolecular charge transfer, and narrows the HOMO-LUMO energy gap, leading to redshifted absorption and fluorescence, as well as reduced fluorescence emission efficiency, thereby significantly improving the PCE by enhancing non-radiative decay channels [39,40,41,42]. We further evaluated the photothermal conversion performance of the four PTAs. The PCE values of BDP 1–4 were determined to be 40.3% ± 1.9%, 45.9% ± 1.4%, 57.8% ± 1.3%, and 88.3% ± 1.2%, respectively (Figure 2c, Table 1). The sequential increase in PCE shows a positive correlation with the enhanced D-A push–pull effect, originating from the progressively strengthened ICT that promotes non-radiative dissipation of excited-state energy. Mechanistically, the enhanced D-A push–pull effect promotes ICT, which competes with radiative decay, thereby quenching fluorescence and converting absorbed energy into heat via non-radiative transitions. Concurrently, the strengthened D-A interaction narrows the HOMO-LUMO energy gap, causing absorption redshift that reduces light scattering and improves tissue penetration. Consequently, from BDP 1 to BDP 4, the progressively stronger D-A effect leads to stepwise redshift, fluorescence quenching, and a stepwise increase in PCE.

Subsequently, we investigated the influence of concentration, laser power, and irradiation time on the temperature rise behavior of BDP 1–4 in DMSO. The results show that under a fixed laser wavelength, the temperature increase is positively correlated with both the photosensitizer concentration (Figure 2d and Figures S1a−S3a) and the laser power (Figure 2e and Figures S1b−S3b), demonstrating good controllability of the photothermal response. After four “laser on-off” cycles, each sample maintained its photothermal heating performance (Figure 2f,g and Figures S1c−S3c), indicating excellent photothermal stability. Under identical test conditions (5 μM, 0.8 W·cm^−2^, 10 min), BDP 4 displayed the most pronounced temperature rise (Figure 2h), which is consistent with its highest PCE. According to the energy gap law, the non-radiative decay rate increases exponentially with decreasing optical band gap (ΔE_opt_) (knr ∝ exp^(−γ∆Eopt⁄ℏwM)^) [43,44,45,46], which theoretically supports the conclusion that BDP 4, with the narrowest energy gap, exhibits the optimal photothermal performance.

To improve water solubility, enhance bioavailability, and increase tumor-site accumulation of the four photothermal agents, we prepared the corresponding nanoparticles (BDP 1–4 NPs) via a nano-co-precipitation method using the amphiphilic polymer DSPE-mPEG_2000_ as the carrier. Transmission electron microscopy (TEM) images indicated that the obtained NPs were uniform in morphology and spherical in shape (Figure S4). Dynamic light scattering (DLS) further revealed that the NPs had a hydrodynamic diameter of approximately 120 nm and a polydispersity index (PDI) in the range of 0.2–0.3. Both the size and dispersity showed no significant change over 7 days (Figure S5), confirming good monodispersity and colloidal stability, which is beneficial for passive targeting via the enhanced permeability and retention (EPR) effect. However, compared with the absorption spectra of the monomers, the encapsulated BDP 1–4 NPs exhibited broadened absorption peaks with the appearance of shoulders (Figure 2i), which may be attributed to the aggregation of BDP molecules (e.g., J- or H-aggregation) within the hydrophobic core of the nanoparticles, as well as changes in microenvironment polarity and conformational restriction—common phenomena in the nanoencapsulation of organic dyes. Furthermore, after encapsulation, all NPs showed significantly weakened fluorescence intensity and a blue shift in emission peaks compared with the monomers (Figure S6), indicating the occurrence of mild aggregation-caused quenching (ACQ). Notably, the nanoparticles could withstand four consecutive laser on/off cycles without significant performance decay (Figures S7c–S10c), demonstrating excellent photothermal stability. The photothermal conversion efficiencies of BDP 1–4 NPs were determined to be 37.6% ±1.4%, 42.5% ± 1.5%, 54.3% ± 1.9%, and 82.8% ± 2.2% (Figure S11), respectively, which are slightly lower than those of their monomers but still remain at a high level, indicating that although encapsulation induces changes in the aggregation state, it does not compromise the excellent photothermal performance. In summary, the optical characterization of BDP 1–4 and their nanoparticles establishes a clear structure–property relationship: a stronger D-A push–pull effect leads to redshifted absorption, weakened fluorescence, and, consequently, a significantly enhanced PCE. These excellent optical properties, particularly the strong near-infrared absorption, high PCE, and outstanding photothermal stability, provide a solid experimental and theoretical basis for their subsequent application in photothermal antitumor therapy at both the cellular and animal levels.

### 2.3. In Vitro Studies

**Figure 3:**
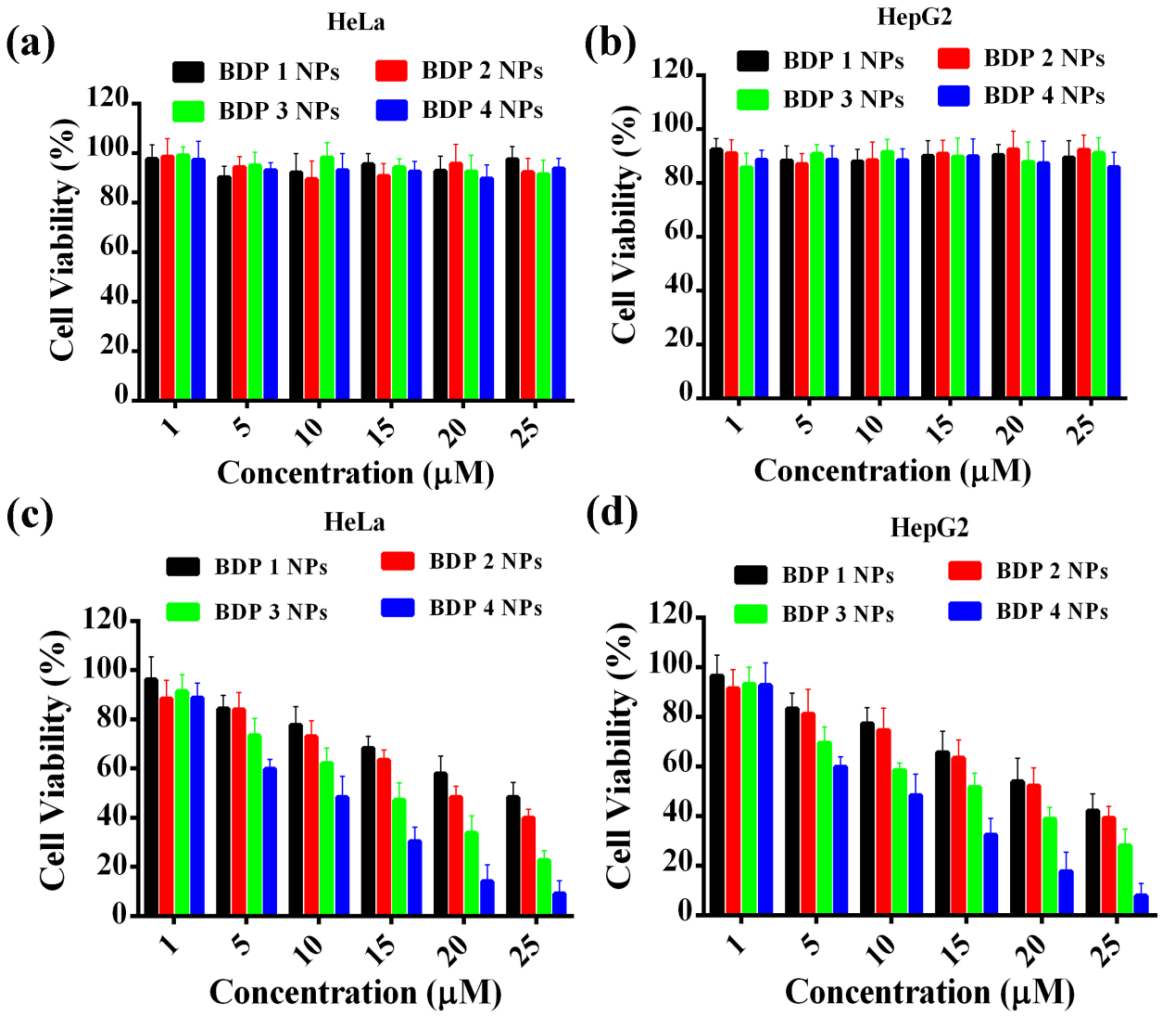
In vitro cytotoxicity evaluation. (**a**,**b**) Dark toxicity of BDP 1–4 NPs against HeLa (**a**) and HepG2 (**b**) cells. (**c**,**d**) Phototoxicity of BDP 1–4 NPs against HeLa (**c**) and HepG2 (**d**) cells under laser irradiation (0.5 W·cm^−2^, 5 min). Data are presented as mean ± SD (*n* = 3).

Here, human cervical adenocarcinoma HeLa and human hepatocellular carcinoma HepG2 cells were used to evaluate the photothermal therapeutic potential of the BDP 1–4 nanoformulations via a standard MTT assay. Both cell lines were treated with various concentrations of BDP 1–4 NPs, with or without subsequent laser irradiation at the corresponding wavelength. As shown in Figure 3, in the absence of laser irradiation, all NP formulations resulted in no significant reduction in cell viability even at the highest tested concentration (25 μM), indicating extremely low dark toxicity and good biocompatibility—a prerequisite for safe in vivo application. However, under low-power laser irradiation (0.5 W·cm^−2^), all NP-treated groups exhibited marked, concentration-dependent photothermal cytotoxicity. The cell-killing efficacy followed the order BDP 4 NPs > BDP 3 NPs > BDP 2 NPs > BDP 1 NPs, which directly correlated with their respective photothermal conversion efficiencies (PCEs). Quantitative analysis further supports this trend. For HeLa cells, the half-maximal inhibitory concentrations (IC_50_) of BDP 1–4 were 24.67, 18.68, 12.48, and 9.66 μM, respectively; for HepG2 cells, the corresponding IC_50_ values were 23.98, 21.56, 16.72, and 8.89 μM. These IC_50_ values decrease progressively with increasing PCE, and BDP 4 nanoparticles (PCE = 88.3%) exhibited the lowest IC_50_ values in both cell lines. At a concentration of 25 μM, they reduced cell viability to approximately 10%, whereas under the same laser irradiation dose, BDP 3 nanoparticles induced approximately 80% cell death at the same concentration. In contrast, BDP 1 and BDP 2 NPs showed comparatively weaker cell killing effects at their respective optimal excitation wavelengths, consistent with their lower PCEs. Taken together, these results demonstrate that PTT efficacy is directly proportional to PCE. BDP 4 NPs, which benefit from the strongest D-A push–pull effect, possess the highest PCE and thereby exhibit optimal photothermal therapeutic performance at the cellular level, supporting their potential for efficient tumor ablation under low-drug-dose and low-laser-power conditions.

### 2.4. In Vivo Studies

**Figure 4:**
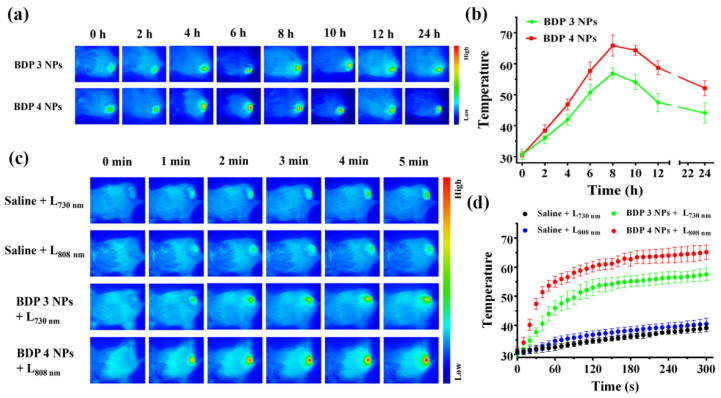
NIR photothermal imaging. (**a**) Time-dependent accumulation of BDP 3 NPs and BDP 4 NPs (200 μM) in tumors over 24 h, monitored by NIR photothermal imaging. (**b**) Quantitative analysis of the data shown in (**a**). (**c**) Comparison of the photothermal effects of BDP 3 NPs and BDP 4 NPs at tumor tissues under continuous laser irradiation. (**d**) Quantitative analysis of the data shown in (**c**). Data are presented as mean ± SD (*n* = 3).

Based on the outstanding in vitro photothermal performance, we further explored the in vivo biological behavior of BDP 3 NPs and BDP 4 NPs in H22 tumor-bearing mouse models. The time-dependent tumor accumulation of both NPs was investigated by photothermal imaging. Briefly, mice with H22 tumors were intravenously injected via the tail vein with BDP 3 NPs, BDP 4 NPs and saline (blank control). At predetermined time points post-injection, the tumor regions were irradiated with the corresponding wavelength laser (0.5 W·cm^−2^) for 5 min, and temperature changes were monitored in real time using a near-infrared thermal camera. As shown in the photothermal images (Figure 4a,b), the tumor temperature peaked at 8 h post-injection in both the BDP 3 NP and BDP 4 NP groups and gradually declined thereafter, indicating maximal NP accumulation in the tumor tissues at this time point. Therefore, all subsequent PTT procedures were performed at 8 h after administration. On this basis, we compared photothermal-induced tissue temperature elevation among different treatment groups. Thermal images (Figure 4c,d) showed that a significant and rapid temperature increase in the tumor area occurred only in the groups that received NP injection followed by corresponding laser irradiation. Among them, the BDP 4 NP group exhibited the most pronounced heating effect, with the local temperature reaching approximately 62 °C, which was noticeably higher than that in the BDP 3 NP group. This result is consistent with the more redshifted absorption, stronger D–A electronic effects, and superior PCE (88.3% vs. 57.8%) of BDP 4, further confirming its enhanced photothermal conversion capability and suggesting its great potential for tumor ablation.

**Figure 5:**
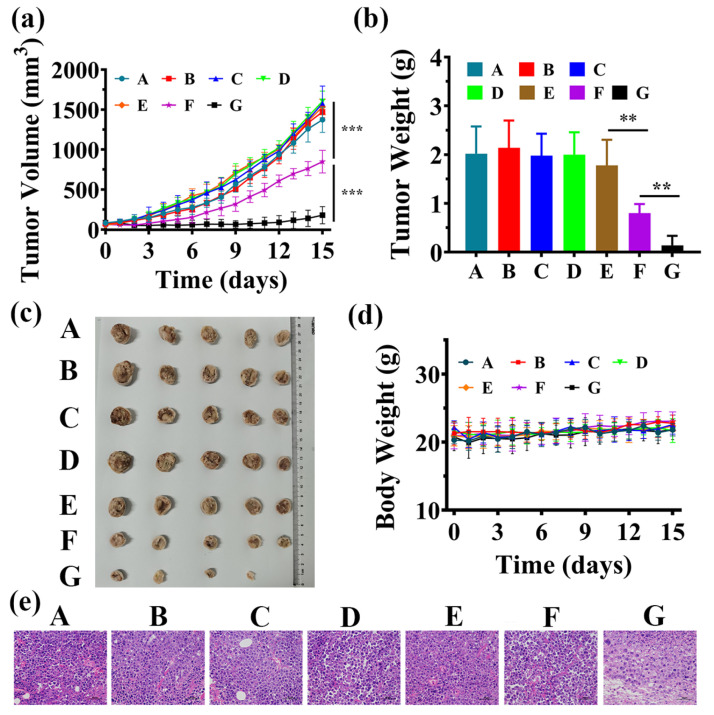
In vivo antitumor activity. (**a**) Tumor volume growth profiles. (**b**) Tumor weights at the experimental endpoint. (**c**) Representative photographs of excised tumors from each group. (**d**) Body weight changes in mice during the treatment period. (**e**) H&E staining of tumor tissues from each treatment group. A: Saline; B: Saline + L_730 nm_; C: Saline + L_808 nm_; D: BDP 3 NPs; E: BDP 4 NPs; F: BDP 3 NPs + L_730 nm_; G: BDP 4 NPs + L_808 nm_. Data are presented as mean ± SD (*n* = 5), *** *p* < 0.001, ** *p* < 0.01.

Next, the in vivo PTT antitumor efficacy of BDP 3 NPs and BDP 4 NPs was assessed using 35 H22 tumor-bearing mice, which were randomly divided into seven groups (*n* = 5 per group): a saline control group (Saline), two laser-only groups (Saline + L_730 nm_ and Saline + L_808 nm_), two nanoparticle-only groups (BDP 3 NPs and BDP 4 NPs), and two nanoparticle-plus-laser treatment groups (BDP 3 NPs + L_730 nm_ and BDP 4 NPs + L_808 nm_). All mice received tail vein injections, followed by laser irradiation (0.5 W·cm^−2^, 5 min) or not at 8 h post-injection. Tumor volume and body weight were measured every other day during the 15-day observation period. As can be seen from the tumor growth curves (Figure 5a), neither NPs alone nor laser irradiation alone effectively suppressed tumor growth. In stark contrast, both NP-plus-laser irradiation groups significantly inhibited tumor growth, with the inhibitory effect of BDP 4 NPs being significantly stronger than that of BDP 3 NPs. The same conclusion was drawn from comparisons of tumor weights (Figure 5b) and representative photographs of excised tumors (Figure 5c) among the seven groups. Meanwhile, the body weights of mice in all groups remained stable throughout the experiment (Figure 5d), suggesting that all formulations, including the combination of NPs with laser treatment, possessed good biosafety in vivo.

To further reveal PTT efficacy, tumors were excised at the end of the experiment and subjected to H&E staining. The results (Figure 5e) revealed that only the BDP 3 NPs + L_730 nm_ and BDP 4 NPs + L_808 nm_ groups displayed obvious pathological damage, such as nuclear fragmentation, with the latter group showing more extensive and severe damage. This indicates that BDP 4 NPs combined with laser excitation induced more pronounced tumor cell necrosis/apoptosis, consistent with its higher PCE compared to BDP 3 NPs. Additionally, histopathological analysis was also performed on major organs (heart, liver, spleen, lung, and kidney). Comparison of H&E-stained sections from the Saline control group, BDP 3 NPs + L_730 nm_ and BDP 4 NPs + L_808 nm_ group (Figure S12) showed no obvious signs of inflammatory infiltration, tissue necrosis, or structural abnormalities in the major organs of mice from the photothermal treatment groups. This result further confirms that both BDP 3 NPs and BDP 4 NPs exhibit good biocompatibility at therapeutic doses without eliciting significant systemic toxicity, providing important safety support for their future applications.

## 3. Materials and Methods

### 3.1. General

The synthesis of BDP-CF_3_ [38], along with solvent purification, instrumental operations, and photophysical/photochemical investigations, are all described in detail in the Electronic Supplementary Materials.

### 3.2. Synthesis

#### 3.2.1. Synthesis of BDP 1

BDP-CF_3_ (0.20 g, 0.64 mmol), 4-(trifluoromethyl)benzaldehyde (1.10 g, 6.32 mmol), activated 4 Å molecular sieves, and anhydrous toluene (50 mL) were placed into a reaction flask. Under a nitrogen atmosphere, piperidine (0.12 mL) and glacial acetic acid (0.10 mL) were added. The mixture was stirred at 80 °C for 2 h. After completion, the reaction mixture was extracted with dichloromethane (50 mL × 3) and water (50 mL). The combined organic layers were dried over anhydrous Na_2_SO_4_. After concentration under reduced pressure, the crude product was purified by silica gel column chromatography using petroleum ether/CH_2_Cl_2_ (2:1, *v*/*v*) as the eluent, yielding BDP 1 as a blue–green solid (0.11 g, 68%). ^1^H NMR (500 MHz, CDCl_3_): δ (ppm) = 7.78 (d, *J* = 16.5 Hz, 2 H), 7.70 (d, *J* = 8.0 Hz, 4 H), 7.64 (d, *J* = 8.0 Hz, 4 H), 7.30 (d, *J* = 16.5 Hz, 2 H), 6.84 (s, 2 H), 2.37 (q, *J* = 3.0 Hz, 6 H). ^13^C NMR (100.6 MHz, CDCl_3_): δ (ppm) = 154.27, 142.30, 139.42, 136.73, 131.11, 130.78, 127.86, 125.87, 125.81, 125.32, 122.61, 121.20, 121.01, 16.00. HRMS (ESI): *m*/*z* calcd for C_30_H_21_BF_11_N_2_ [M + H]^+^: 629.1617, found 629.1604.

#### 3.2.2. Synthesis of BDP 2

Following a procedure similar to that described for BDP 1, treatment of BDP-CF_3_ (0.10 g, 0.32 mmol) with benzaldehyde (0.55 g, 3.16 mmol) in the presence of piperidine (0.12 mL) and glacial acetic acid (0.10 mL) gave BDP 2 as a blue–green solid (0.14 g, 35%). ^1^H NMR (500 MHz, CDCl_3_): δ (ppm) = 7.75 (d, *J* = 16.5 Hz, 2 H), 7.64 (d, *J* = 7.5 Hz, 4 H), 7.43–7.40 (m, 4 H), 7.37–7.32 (m, 4 H), 6.83 (s, 2 H), 2.38 (q, *J* = 3.0 Hz, 6 H). ^13^C NMR (100.6 MHz, CDCl_3_): δ (ppm) = 154.62, 141.53, 138.58, 136.24, 133.92, 129.60, 128.90, 127.89, 124.01, 121.32, 120.88, 118.95, 16.00. HRMS (ESI): *m*/*z* calcd for C_28_H_23_BF_5_N_2_ [M + H]^+^: 493.1869, found 493.1855.

#### 3.2.3. Synthesis of BDP 3

According to the general procedure described for BDP 1, BDP-CF_3_ (0.10 g, 0.32 mmol) was reacted with 4-hydroxybenzaldehyde (0.19 g, 1.6 mmol), piperidine (0.12 mL), and glacial acetic acid (0.10 mL). Purification by silica gel column chromatography (CH_2_Cl_2_/MeOH = 15:1, *v*/*v*) afforded BDP 3 as a grassy-green solid (0.12 g, 75%). ^1^H NMR (400 MHz, DMSO-d_6_): δ (ppm) = 10.15 (s, 2 H), 7.65 (d, *J* = 16.0 Hz, 2 H), 7.52 (d, *J* = 8.0 Hz, 4 H), 7.37 (d, *J* = 16.8 Hz, 2 H), 7.23 (s, 2 H), 6.89 (d, *J* = 8.0 Hz, 4 H), 2.32 (s, 6 H). ^13^C NMR (100.6 MHz, DMSO-d_6_): δ (ppm) = 162.64, 159.84, 154.38, 140.05, 139.94, 132.66, 129.73, 127.21, 123.97, 121.46, 116.27, 114.65, 15.38. HRMS (ESI): *m*/*z* calcd for C_28_H_21_BF_5_N_2_O_2_ [M + K]^+^: 563.1326, found 563.1341.

#### 3.2.4. Synthesis of BDP 4

Following a procedure analogous to that for BDP 1, the reaction was carried out except that 4-(trifluoromethyl)benzaldehyde was replaced by 4-(*N*,*N*-dimethylamino)benzaldehyde. The crude product was purified by silica gel column chromatography using petroleum ether/CH_2_Cl_2_ (1:1, *v*/*v*) as the eluent, yielding BDP 4 as a brownish-black solid (0.21 g, 74%). ^1^H NMR (400 MHz, CDCl_3_): δ (ppm) = 7.59–7.34 (m, 6 H), 7.28–7.24 (m, 2 H), 6.78–6.74 (m, 6 H), 3.06 (s, 12 H), 2.34 (s, 6 H).

### 3.3. In Vitro Studies

#### 3.3.1. Measurement of Electronic Absorption and Fluorescence Spectra

Stock solutions of BDP 1–4 (1 mM) were first prepared by dissolving the corresponding compounds in DMSO. Subsequently, 10 μM sample solutions were prepared by diluting the stock solutions with the appropriate solvent for UV–vis absorption and fluorescence emission measurements. All spectral data were processed and fitted using Origin 8.0 software.

#### 3.3.2. Photothermal Effect

To evaluate the photothermal conversion performance of BDP 1–4, the effect of laser power density on temperature rise was first investigated. Specifically, sample solutions of BDP 1–4 (10 μM in DMSO) were irradiated continuously for 10 min with an 808 nm laser at power densities of 0.2, 0.4, 0.6, 0.8, and 1.0 W·cm^−2^, and the temperature changes were monitored in real time using an infrared thermal camera (Shanghai Thermal Imaging Technology Co., Ltd., Shanghai, China). Next, the concentration-dependence temperature rises were examined. Subsequently, the concentration-dependent temperature increase was examined. BDP 1–4 were dissolved in DMSO at concentrations of 0, 1, 2.5, 5, and 10 μM and irradiated continuously for 10 min with an 808 nm laser at a fixed power density of 1.0 W·cm^−2^. The corresponding temperature rise profiles were recorded.

#### 3.3.3. Evaluation of Photothermal Conversion Efficiency

The PCE was determined using the following procedure: Two types of sample solutions (BDP 1–4 in DMSO and BDP 1–4 NPs in PBS) were prepared at a concentration of 5 μM. Each solution was irradiated separately with 660 nm, 660 nm, 730 nm, and 808 nm lasers at a power density of 0.8 W·cm^−2^ for 10 min, followed by natural cooling to room temperature. Temperature changes during irradiation were monitored in real time using a near-infrared thermal imaging camera. Each experiment was performed in triplicate, and the results are reported as mean ± standard deviation.

The PCE (*η*) was calculated according to the literature method [47], using the following equation:

$$\eta =\frac{hs\left(T_{max}-T_{surr}\right)-Q_{0}}{I(1-10^{{-A}_{\lambda }})}$$

Here, *h* is the heat transfer coefficient, *s* is the surface area of the container, *Q*_0_ is the heat generated by the solvent, *I* is the laser power, and *A_λ_* is the absorbance at the excitation wavelength. The heat transfer term *hs* is derived from the cooling curve using:

$$hs=\frac{mc}{\tau_{s}}$$

where *m* is the mass of the solution, *c* is its specific heat capacity, and *τ_s_* is the time constant obtained by fitting the linearized cooling curve:

$$t=-\tau_{s}\;Ln(\theta )$$

The dimensionless temperature *θ* is defined as follows:

$$\theta =\frac{T-T_{surr}}{T_{max}-T_{surr}}$$

where *T* is the current temperature, *T_max_* is the highest steady-state temperature, and *T_surr_* is the ambient temperature.

#### 3.3.4. Preparation of Nanoparticles

BDP 1–4 NPs were prepared by using the nanoprecipitation method. Briefly, 2 mg of each compound (BDP 1–4) was separately dissolved in 1.5 mL of tetrahydrofuran. Meanwhile, 25 mg of DSPE-mPEG_2000_ was dissolved in 10 mL of deionized water and sonicated for 5 min to ensure complete dissolution. Under magnetic stirring, the organic phase was added dropwise into the aqueous phase, and the mixture was stirred at room temperature for 12 h. The resulting suspension was then transferred to a 50 mL ultrafiltration centrifuge tube and washed by centrifugation at 3500 rpm for 2–3 cycles. The collected nanoparticle dispersion was filtered through a 0.22 μm membrane to obtain BDP 1–4 NPs, which were stored at 4 °C for further use. The morphology and size of the nanoparticles were characterized by transmission electron microscopy (Thermo Fisher Scientific, Hillsboro, OR, USA).

#### 3.3.5. Cytotoxicity Assessment of Photothermal Agents

The in vitro cytotoxicity of BDP 1–4 NPs was evaluated using human cervical carcinoma HeLa and human hepatocellular carcinoma HepG2 cells as models via the standard MTT assay. Briefly, cells were seeded in 96-well plates, cultured for 12 h, and then treated with varying concentrations of nanoparticles (1, 5, 10, 15, 20, and 25 μM) for 24 h. After removing the culture medium, the experimental groups were irradiated with an 808 nm laser (0.5 W·cm^−2^) for 5 min, while the control groups were kept in the dark. Following overnight incubation, the MTT solution was added and the cells were incubated for an additional 4 h. The supernatant was then discarded, and DMSO was added to dissolve the formazan crystals. Absorbance was measured at 517 nm to determine cell viability.

### 3.4. In Vivo Studies

#### 3.4.1. In Vivo Photothermal Imaging Evaluation

A total of twelve 5-to-6-week-old female BALB/c mice were employed to establish subcutaneous xenograft tumor models via injection of H22 cells. When the tumor volume reached approximately 100–150 mm^3^, the mice were randomly divided into four groups (*n* = 3 per group) and intravenously injected via the tail vein with BDP 3 NPs (200 μM), BDP 4 NPs (200 μM), or an equal volume of saline (200 μL). At 8 h post-injection, the tumor regions were irradiated with laser of the corresponding wavelengths (BDP 3 NPs: 730 nm; BDP 4 NPs: 808 nm) at a power density of 0.5 W·cm^−2^ for 5 min. Throughout the procedure, temperature changes in the tumor area of the mice were monitored using a near-infrared thermal camera (Shanghai Thermal Imaging Technology Co., Ltd., Shanghai, China). 

#### 3.4.2. In Vivo Antitumor Efficacy and Biosafety Evaluation

H22 tumor-bearing mouse models (*n* = 35) were established according to the protocol described above. When the tumor volume reached 60–100 mm^3^, the mice were randomly divided into seven groups (*n* = 5 per group): a saline control group (Saline), laser-only groups (Saline + L_730 nm_/Saline + L_808 nm_), nanoparticle-only groups (BDP 3 NPs and BDP 4 NPs), and nanoparticle-plus-laser treatment groups (BDP 3 NPs + L_730 nm_ and BDP 4 NPs + L_808 nm_). Each mice in the seven group received a corresponding formulation via tail vein injection (200 μL per mouse). At 8 h post-injection, the tumor tissues of the mice in the light-irradiated groups were exposed to laser irradiation at the respective wavelength (0.5 W·cm^−2^, 5 min). During the 15-day treatment period, tumor volume and mouse body weight were measured every two days. At the experimental endpoint, the mice were euthanized; tumors were excised and weighed, and major organs (heart, liver, spleen, lung, and kidney) and tumor tissues were collected. Tissues were fixed in 4% paraformaldehyde, embedded in paraffin, sectioned, and subjected to hematoxylin and eosin (H&E) staining for histopathological analysis.

## 4. Conclusions

In this study, we designed and synthesized a series of BODIPY-based derivatives (BDP 1–4) featuring gradient D-A push–pull electronic effects for highly efficient photothermal antitumor therapy. Their structure–activity relationships were systematically elucidated through photophysical characterization and in vitro/in vivo photobiological evaluation. From BDP 1 to BDP 4, the progressively strengthened push–pull effect led to enhanced intramolecular charge transfer, a narrowed HOMO-LUMO gap, redshifted absorption, weakened fluorescence emission, and a remarkable increase in photothermal conversion efficiency up to 88.3%. This PCE value is outstanding compared to typical values (40–60%) reported for BODIPY-based photothermal agents. To improve water solubility and tumor targeting, these molecules were further encapsulated into nanoparticles (NPs) by DSPE-PEG_2000_. The as-prepared BDP 1–4 NPs remained stable for at least 7 days at room temperature and withstood four consecutive laser on/off cycles without losing their photothermal performance. Both in vitro and in vivo studies demonstrated that under low-power laser irradiation (0.5 W·cm^−2^), the nanoformulation of BDP 4 with the highest PCE among the series achieved pronounced photothermal tumor ablation without inducing systemic toxicity. This study proposes a molecular design strategy that synergistically modulates near-infrared absorption and photothermal conversion by enhancing the D-A push–pull effect. This strategy provides a design rationale for developing efficient and low-toxicity organic photothermal agents, demonstrates its potential applicability in low-power photothermal therapy modalities, and may serve as a reference for further research on precision photothermal therapy of tumors. A limitation of this study is that the efficacy against deep-seated or metastatic tumors requires further validation, as photothermal therapy is inherently local and the 808 nm light used has limited tissue penetration depth. To address this limitation, future efforts could focus on developing near-infrared II (NIR-II) organic photothermal agents for deeper tissue penetration, or on combining photothermal therapy with immunotherapy or other treatment modalities to achieve synergistic effects against metastatic tumors.

## Figures and Tables

**Table 1:** Optical properties of BDP 1–4 in DMSO.

Compound	$\lambda_{max}^{abs}$ (nm)	ε (L mol^−1^ cm^−1^)	$\lambda_{max}^{em}$ (nm)	Ф_F_	η (%)
BDP 1	691	100,110	735	0.05	40.3 ± 1.9
BDP 2	699	94,400	744	0.044	45.9 ± 1.4
BDP 3	742	98,800	800	0.033	57.8 ± 1.3
BDP 4	843	95,410	N.D.	0.001	88.3 ± 1.2

ε: Molar extinction coefficient; Ф_F_: fluorescence quantum yield; η: photothermal conversion efficiency.

## Data Availability

The authors declare that all the data obtained in this study can be provided on request.

## Supplementary Materials

The following supporting information can be downloaded at: https://www.mdpi.com/article/10.3390/molecules31132258/s1, Figure S1. Photothermal characterization of BDP 1; Figure S2. Photothermal characterization of BDP 2; Figure S3. Photothermal characterization of BDP 4; Figure S4. DLS size distribution and TEM images of BDP 1, BDP 2, and BDP 3; Figure S5. Changes in particle size and PDI of BDP 1–4 over a 7-day period; Figure S6. Fluorescence emission spectra of BDP 1–4 NPs at a concentration of 10 μM; Figure S7. Evaluation of photothermal conversion efficiency of BDP 1 NPs; Figure S8. Evaluation of photothermal conversion efficiency of BDP 2 NPs; Figure S9. Evaluation of photothermal conversion efficiency of BDP 3 NPs; Figure S10. Evaluation of photothermal conversion efficiency of BDP 4 NPs; Figure S11. Comparison of PCE among BDP 1–4 NPs; Figure S12. Biosafety evaluation: H&E staining of major organs after various treatments under different conditions. Figure S13. ^1^H NMR spectrum of BDP-CF_3_ in CDCl_3_. Figure S14. ^1^H NMR spectrum of BDP 1 in CDCl_3_. Figure S15. ^13^C NMR spectrum of BDP 1 in CDCl_3_. Figure S16. HRMS spectrum of BDP 1. Figure S17. ^1^H NMR spectrum of BDP 2 in CDCl_3_. Figure S18. ^13^C NMR spectrum of BDP 2 in CDCl_3_. Figure S19. HRMS spectrum of BDP 2. Figure S20. ^1^H NMR spectrum of BDP 3 in DMSO-d_6_. Figure S21. ^13^C NMR spectrum of BDP 3 in DMSO-d_6_. Figure S22. HRMS spectrum of BDP 3. Figure S23. ^1^H NMR spectrum of BDP 4 in DMSO-d_6_. Table S1. Optical properties of BDP 1-4 NPs.

## Abbreviations

The following abbreviations are used in this manuscript:

PCE	photothermal conversion efficiency
PTAs	photothermal agents
BODIPY	boron-dipyrromethene
PTT	photothermal therapy
NIR	near-infrared
D-A	donor–acceptor
ICT	intramolecular charge transfer
EPR	enhanced permeability and retention
HRMS	high-resolution mass spectrometry
