# Supplementary material for: Strengthening D-A Push–Pull Interactions in BODIPY to Enhance Near-Infrared Absorption and Photothermal Conversion for Low-Intensity Photothermal Antitumor Therapy
Source: Molecules. 2026 Jun 26;31(13):2258. doi: 10.3390/molecules31132258 (PMC13363352; doi:10.3390/molecules31132258)
Supplement: Supplementary file 1 [file molecules-31-02258-s001.zip › molecules-4347967-supplementary.pdf]

Electronic Supporting Information

Strengthening D-A Push–Pull Interactions in BODIPY to Enhance Near-Infrared Absorption and Photothermal Conversion for Low-Intensity Photothermal Antitumor Therapy

Yamin Li, Xiaolu Weng and Jianyong Liu *

Key Laboratory of Molecule Synthesis and Function Discovery, Fujian Province University, College of
Chemistry, Fuzhou University, Fuzhou 350108, China; 15236954674@163.com (Y.L.);
15213509135@163.com (X.W.)
* Correspondence: lkw82@fzu.edu.cn

Table of Contents

General

Synthesis of BDP-CF_3

Figure S1. Photothermal characterization of **BDP 1**: (a) Temperature elevation curves of **BDP 1** at different concentrations under 660 nm laser irradiation (0.8 W·cm^{-2}). (b) Temperature elevation curves of **BDP 1** (5 μM) under different laser power densities. (c) Temperature variation of **BDP 1** over four heating/cooling cycles under 660 nm laser irradiation. (d) Linear relationship between -Ln(θ) and time for **BDP 1**.

Figure S2. Photothermal characterization of **BDP 2**: (a) Temperature elevation curves of **BDP 2** at different concentrations under 660 nm laser irradiation (0.8 W·cm^{-2}). (b) Temperature elevation curves of **BDP 2** (5 μM) under different laser power densities. (c) Temperature variation of **BDP 2** over four heating/cooling cycles under 660 nm laser irradiation. (d) Linear relationship between -Ln(θ) and time for **BDP 2**.

Figure S3. Photothermal characterization of **BDP 4**: (a) Temperature elevation curves of **BDP 4** at different concentrations under 808 nm laser irradiation (0.8 W·cm^{-2}). (b) Temperature elevation curves of **BDP 4** (5 μM) under different laser power densities. (c) Temperature variation of **BDP 4** over four heating/cooling cycles under 808 nm laser irradiation. (d) Linear relationship between -Ln(θ) and time for **BDP 4**.

Figure S4. DLS size distribution and TEM images of **BDP 1 NPs**, **BDP 2 NPs**, **BDP 3 NPs** and **BDP 4 NPs**.

Figure S5. Changes in particle size and PDI of **BDP 1-4 NPs** over a 7-day period.

Figure S6. Fluorescence emission spectra of **BDP 1-4 NPs** at a concentration of 10 μM.

Figure S7. Photothermal characterization of **BDP 1 NPs**: (a) Temperature elevation curves of **BDP 1 NPs** at different concentrations under 660 nm laser irradiation (0.8 W·cm^{-2}). (b) Temperature elevation curves of **BDP 1 NPs** (5 μM) under different laser power densities. (c) Temperature variation of **BDP 1 NPs** over four heating/cooling cycles under 660 nm laser irradiation. (d) Linear relationship between -Ln(θ) and time for **BDP 1 NPs**.

Figure S8. Photothermal characterization of **BDP 2 NPs**: (a) Temperature elevation curves of **BDP 2 NPs** at different concentrations under 660 nm laser irradiation (0.8 W·cm^{-2}). (b) Temperature elevation curves of **BDP 2 NPs** (5 μM) under different laser power densities. (c) Temperature variation of **BDP 2 NPs** over four heating/cooling cycles under 660 nm laser irradiation. (d) Linear relationship between -Ln(θ) and time for **BDP 2 NPs**.

Figure S9. Photothermal characterization of **BDP 3 NPs**: (a) Temperature elevation curves of **BDP 3 NPs** at different concentrations under 730 nm laser irradiation (0.8 W·cm^{-2}).

(b) Temperature elevation curves of **BDP 3 NPs** (5 μM) under different laser power densities. (c) Temperature variation of **BDP 3 NPs** over four heating/cooling cycles under 730 nm laser irradiation. (d) Linear relationship between -Ln(θ) and time for **BDP 3 NPs**.

Figure S10. Photothermal characterization of **BDP 4 NPs**: (a) Temperature elevation curves of **BDP 4 NPs** at different concentrations under 808 nm laser irradiation (0.8 $W \cdot cm^{-2}$). (b) Temperature elevation curves of **BDP 4 NPs** (5 μM) under different laser power densities. (c) Temperature variation of **BDP 4 NPs** over four heating/cooling cycles under 808 nm laser irradiation. (d) Linear relationship between -Ln(θ) and time for **BDP 4 NPs**.

Figure S11. Comparison of PCE among **BDP 1-4 NPs**: (a) Comparison of temperature variation profiles of **BDP 1-4 NPs** under a laser irradiation and cooling cycle. (b) Comparison of PCE among **BDP 1-4 NPs**.

Table S1. Optical properties of **BDP 1-4 NPs**

Figure S12. Biosafety evaluation: H&E staining of major organs after various treatments under different conditions

Figure S13. ^{1}H NMR spectrum of **BDP-CF$_3$** in $CDCl_3$

Figure S14. ^{1}H NMR spectrum of **BDP 1** in $CDCl_3$

Figure S15. ^{13}C NMR spectrum of **BDP 1** in $CDCl_3$

Figure S16. HRMS spectrum of **BDP 1**

Figure S17. ^{1}H NMR spectrum of **BDP 2** in $CDCl_3$

Figure S18. ^{13}C NMR spectrum of **BDP 2** in $CDCl_3$

Figure S19. HRMS spectrum of **BDP 2**

Figure S20. ^{1}H NMR spectrum of **BDP 3** in DMSO-d_6

Figure S21. ^{13}C NMR spectrum of **BDP 3** in DMSO-d_6

Figure S22. HRMS spectrum of **BDP 3**

Figure S23. ^{1}H NMR spectrum of **BDP 4** in $CDCl_3$

General

Reagents

Toluene and dichloromethane (CH_2Cl_2) were distilled over sodium and calcium hydride, respectively. All other reagents were obtained from commercial suppliers without further purification. All reactions were performed under a nitrogen atmosphere.

Apparatus

^1H NMR spectra were measured on an AVANCE III 400 spectrometer (^1H, 400 MHz) and AVANCE III 500 (^1H, 500 MHz) spectrometer (Bruker, Karlstuhe, Germany), and ^{13}C NMR spectra were recorded on an AVANCE III 600 (^{13}C, 151 MHz) spectrometer (Bruker, Karlstuhe, Germany) at ambient temperatures. $CDCl_3$ and DMSO-d_6 were utilized as the solvents, and chemical shifts are expressed in parts per million (ppm) relative to tetramethylsilane (TMS, $\delta = 0$ ppm). High-resolution mass spectrometry (HRMS) was performed using the Agilent 6520 Accurate-Mass Q-TOF Mass Spectrometer (Agilent Technologies, Santa Clara, CA, USA) in positive mode with electrospray ionization (ESI). Electronic absorption and fluorescence emission spectra were obtained using a PerkinElmer Lambda 365 UV-Visible Absorption Spectrometer (Perking Elmer, Waltham, MA, USA) and a VARIAN Carye Elipse Fluorescence Spectrometer (Agilent Technologies, Santa Clara, CA, USA), respectively. We performed thermal imaging on small animals using a preclinical in vivo imaging system from PerkinElmer Inc. (Waltham, Massachusetts, USA). All cell lines were purchased from the Cell Bank of the Shanghai Institute of Biochemistry and Cell Biology, Chinese Academy of Sciences (Shanghai, China). Mice were supplied by Wushi Experimental Animal Trading Co., Ltd. (Minhou County, China).

Synthesis of BDP-CF_3

Trifluoroacetic acid (2.0 mL, 1.0 equiv) was dissolved in anhydrous CH_2Cl_2 (100 mL) under a nitrogen atmosphere. Phenyltrichlorosilane (2.0 mL, 2.0 equiv) was added dropwise to the stirred solution over 10 minutes while cooling in an ice-water bath. The mixture was stirred for an additional 20 minutes at 0 °C. In a separate round-bottom flask, a mixture of 2,4-dimethylpyrrole (2.0 mL, 2.0 equiv) and triethylamine (5.7 mL, 6.0 equiv) in CH_2Cl_2 (20 mL) was stirred at room temperature for 10 minutes. This mixture was then transferred to the main reaction vessel containing the acid/silane solution and stirred in the ice bath for 30 minutes, during which the color changed to bluish-green. Boron trifluoride diethyl etherate (6.0 mL, 5.0 equiv) was added dropwise at 0°C. The reaction was then allowed to warm to room temperature and stirred overnight. Upon completion, the mixture was diluted with CH_2Cl_2 (100 mL) and washed with water (100 mL × 3). The combined organic layers were dried over anhydrous Na_2SO_4 and concentrated under reduced pressure. The crude product was purified by silica gel column chromatography using petroleum ether/CH_2Cl_2 (5:2, v/v) as the eluent to afford BDP-CF_3 as a golden-yellow solid (0.41 g, 13%). ^1H NMR (400 MHz,

$CDCl_3$): δ 6.15 (s, 1 H), 2.54 (s, 2 H), 2.30 (s, 2 H).

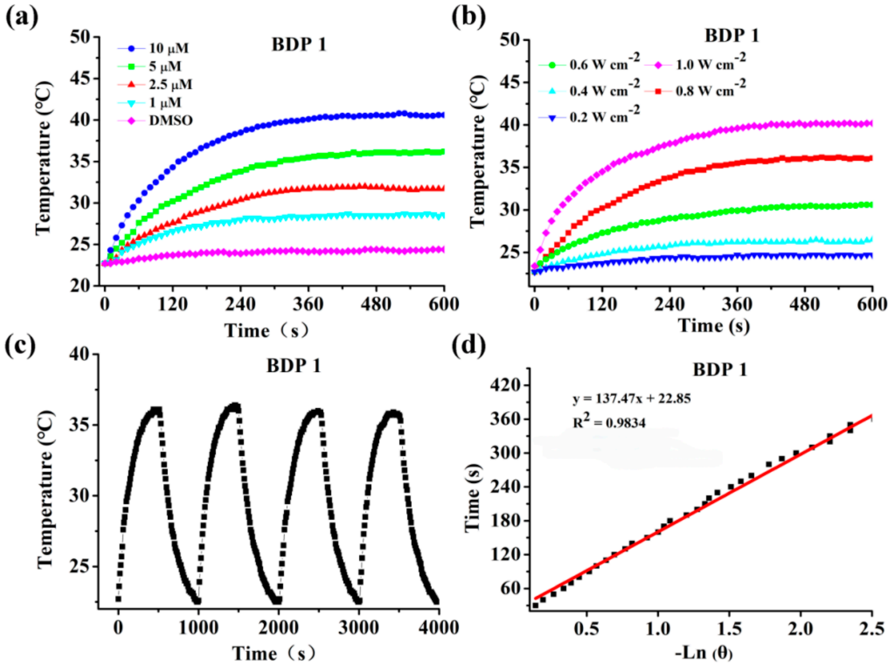

Figure S1. Photothermal characterization of **BDP 1**: (a) Temperature elevation curves of **BDP 1** at different concentrations under 660 nm laser irradiation (0.8 W·cm⁻²). (b) Temperature elevation curves of **BDP 1** (5 μM) under different laser power densities. (c) Temperature variation of **BDP 1** over four heating/cooling cycles under 660 nm laser irradiation. (d) Linear relationship between -Ln(θ) and time for **BDP 1**.

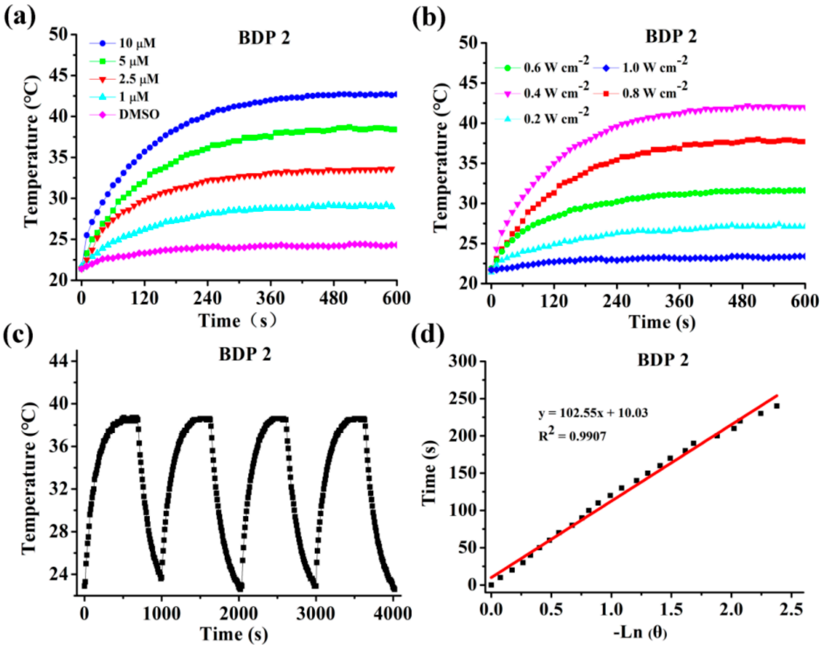

Figure S2. Photothermal characterization of **BDP 2**: (a) Temperature elevation curves of **BDP 2** at different concentrations under 660 nm laser irradiation (0.8 W·cm⁻²). (b) Temperature elevation curves of **BDP 2** (5 μM) under different laser power densities. (c) Temperature variation of **BDP 2** over four heating/cooling cycles under 660 nm laser irradiation. (d) Linear relationship between -Ln(θ) and time for **BDP 2**.

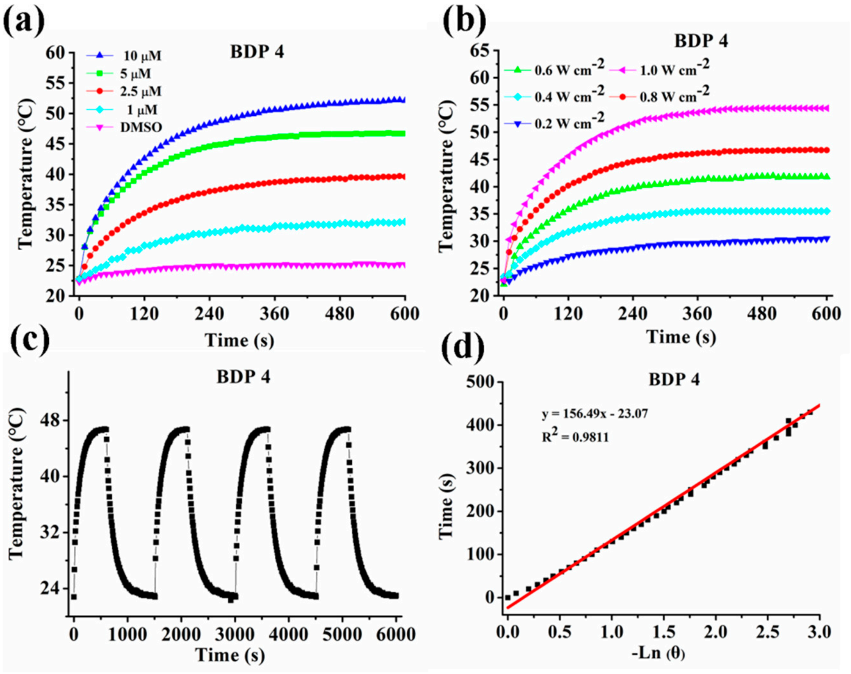

Figure S3. Photothermal characterization of **BDP 4**: (a) Temperature elevation curves of **BDP 4** at different concentrations under 808 nm laser irradiation (0.8 W·cm^{-2}). (b) Temperature elevation curves of **BDP 4** (5 μM) under different laser power densities. (c) Temperature variation of **BDP 4** over four heating/cooling cycles under 808 nm laser irradiation. (d) Linear relationship between -Ln(θ) and time for **BDP 4**.

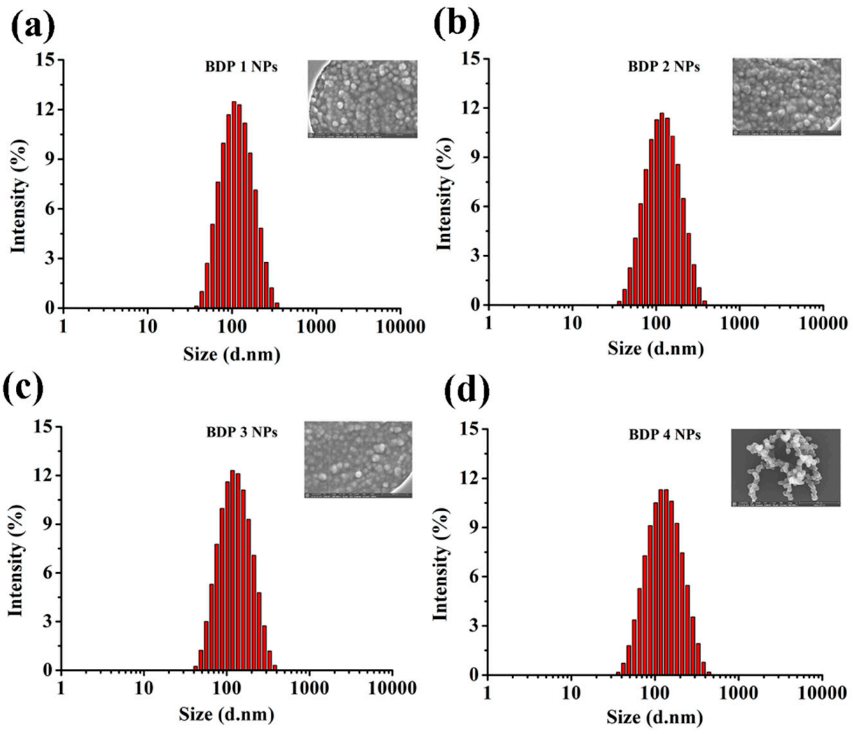

Figure S4. DLS size distribution and TEM images of **BDP 1 NPs**, **BDP 2 NPs**, **BDP 3 NPs** and **BDP 4 NPs**. Scale bar: 500 nm.

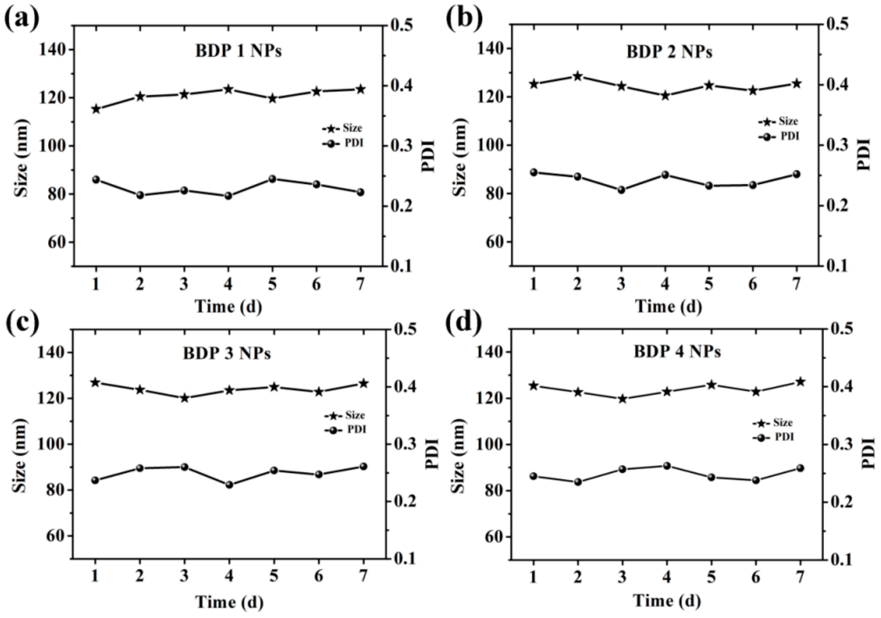

Figure S5. Changes in particle size and PDI of **BDP 1-4 NPs** over a 7-day period.

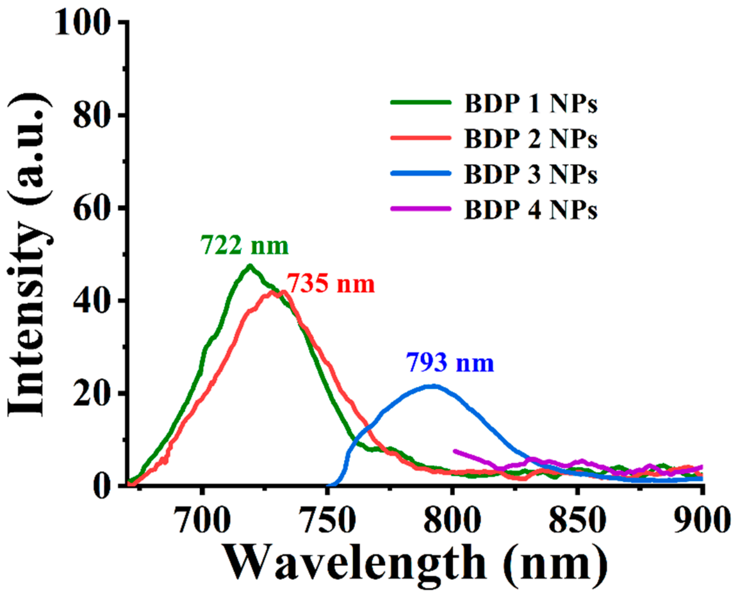

Figure S6. Fluorescence emission spectra of **BDP 1-4 NPs** at a concentration of 10 μM.

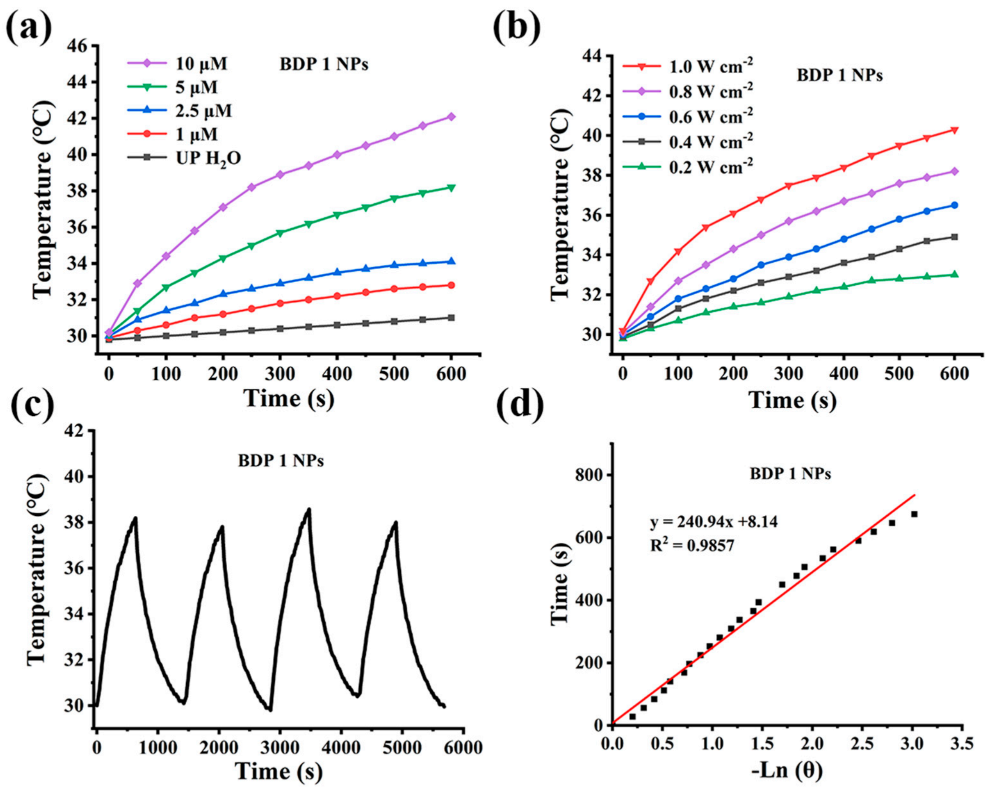

Figure S7. Photothermal characterization of **BDP 1 NPs**: (a) Temperature elevation curves of **BDP 1 NPs** at different concentrations under 660 nm laser irradiation (0.8 W·cm⁻²). (b) Temperature elevation curves of **BDP 1 NPs** (5 μM)

under different laser power densities. (c) Temperature variation of **BDP 1 NPs** over four heating/cooling cycles under 660 nm laser irradiation. (d) Linear relationship between -Ln(θ) and time for **BDP 1 NPs**.

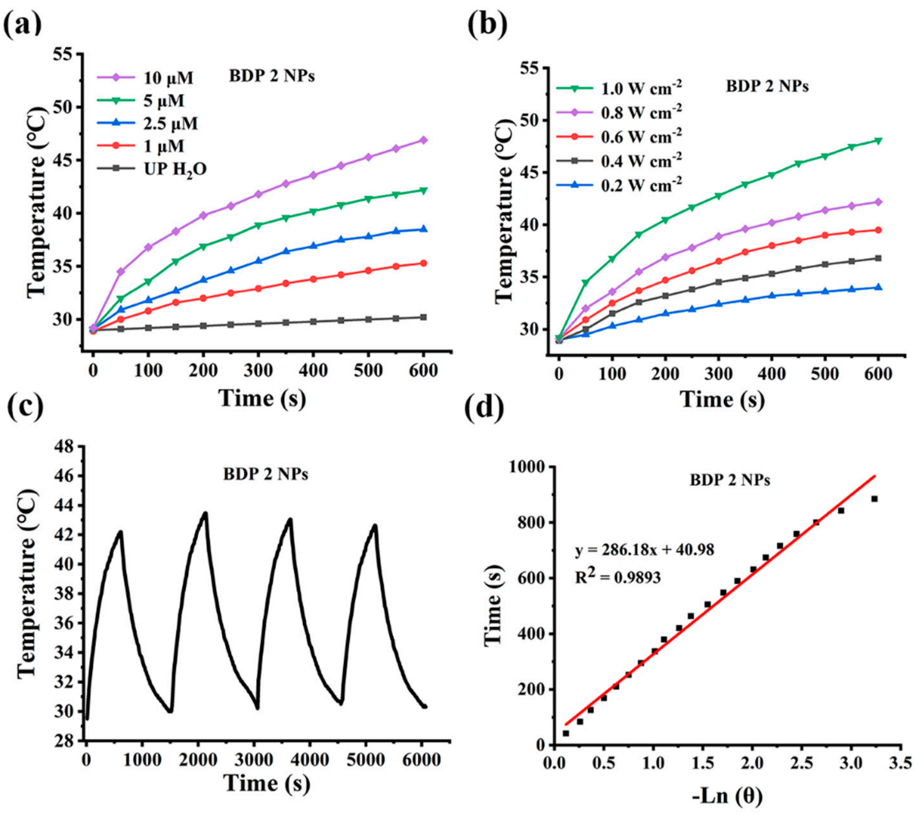

Figure S8. Photothermal characterization of **BDP 2 NPs**: (a) Temperature elevation curves of **BDP 2 NPs** at different concentrations under 660 nm laser irradiation (0.8 W·cm^{-2}). (b) Temperature elevation curves of **BDP 2 NPs** (5 μM) under different laser power densities. (c) Temperature variation of **BDP 2 NPs** over four heating/cooling cycles under 660 nm laser irradiation. (d) Linear relationship between -Ln(θ) and time for **BDP 2 NPs**.

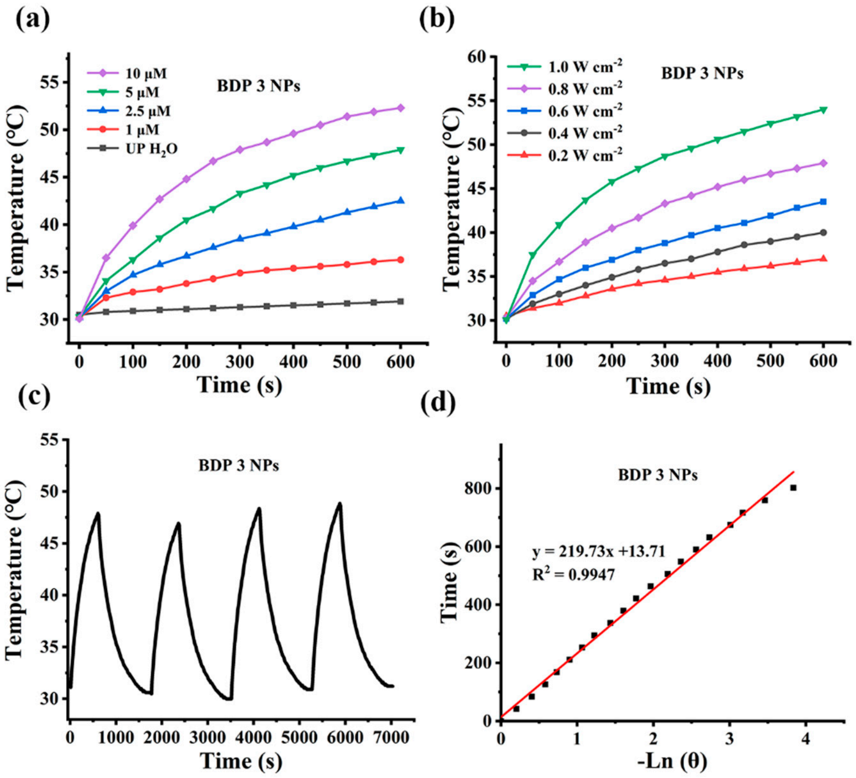

Figure S9. Photothermal characterization of **BDP 3 NPs**: (a) Temperature elevation curves of **BDP 3 NPs** at different concentrations under 730 nm laser irradiation (0.8 W·cm⁻²). (b) Temperature elevation curves of **BDP 3 NPs** (5 μM) under different laser power densities. (c) Temperature variation of **BDP 3 NPs** over four heating/cooling cycles under 730 nm laser irradiation. (d) Linear relationship between -Ln(θ) and time for **BDP 3 NPs**.

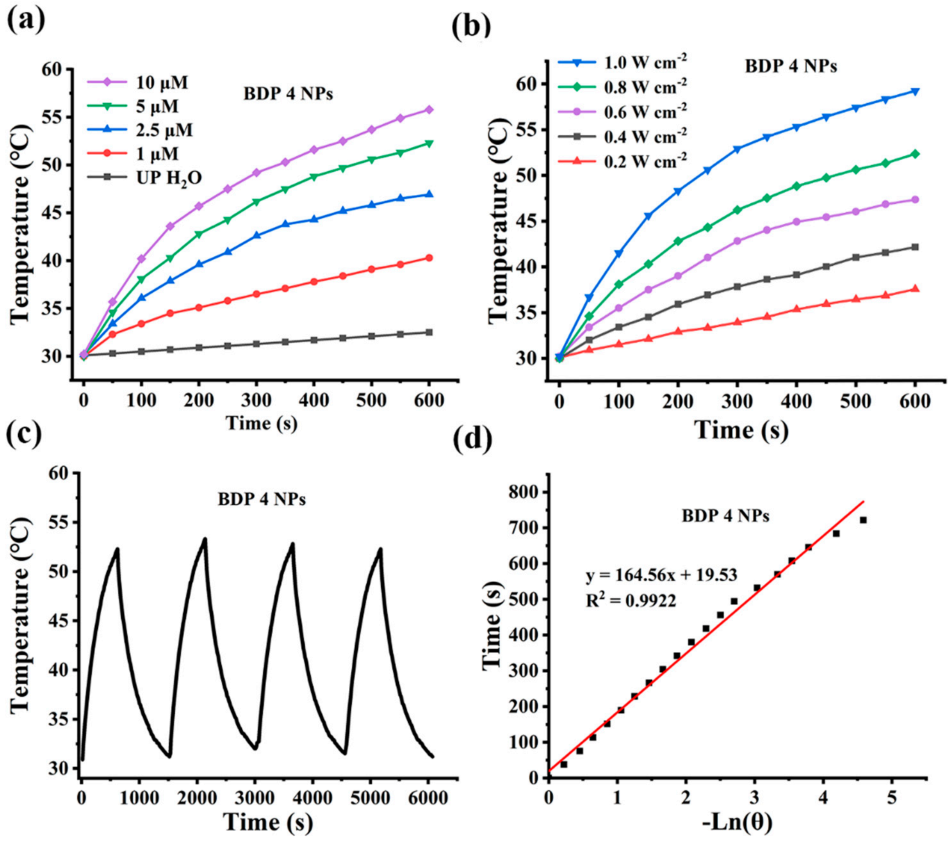

Figure S10. Photothermal characterization of **BDP 4 NPs**: (a) Temperature elevation curves of **BDP 4 NPs** at different concentrations under 808 nm laser irradiation (0.8 W·cm^{-2}). (b) Temperature elevation curves of **BDP 4 NPs** (5 μM) under different laser power densities. (c) Temperature variation of **BDP 4 NPs** over four heating/cooling cycles under 808 nm laser irradiation. (d) Linear relationship between -Ln(θ) and time for **BDP 4 NPs**.

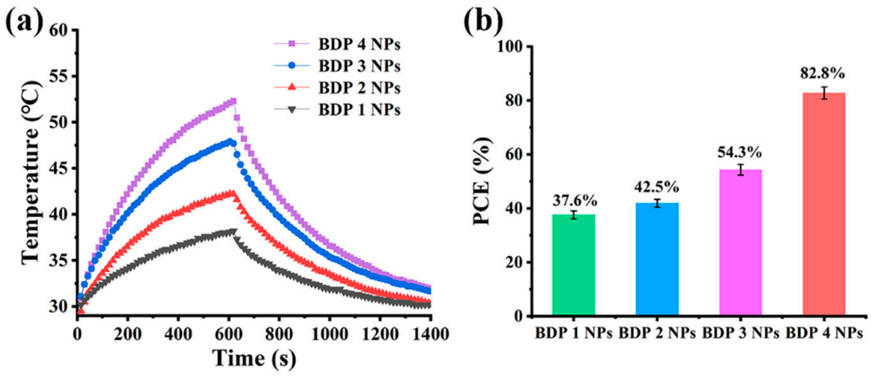

Figure S11. (a) Comparison of temperature variation profiles of **BDP 1-4 NPs** under a laser irradiation and cooling cycle. (b) Comparison of PCE among **BDP 1-4 NPs**. Data are presented as mean ± SD (n=3).

Table S1. Optical properties of **BDP 1-4 NPs.**

Compound	λ_{max}^{abs} (nm)	ε (L mol^{-1} cm^{-1})	λ_{max}^{em} (nm)	Φ_F	η (%)
BDP 1 NPs	700	100030	722	0.031	37.6±1.4
BDP 2 NPs	723	93600	735	0.034	42.5±1.5
BDP 3 NPs	765	98320	793	0.028	54.3±1.9
BDP 4 NPs	856	95270	N.D.	N.D.	82.8±2.2

ε: Molar extinction coefficient; Φ_F: Fluorescence quantum yield; η: Photothermal conversion effi-ciency;

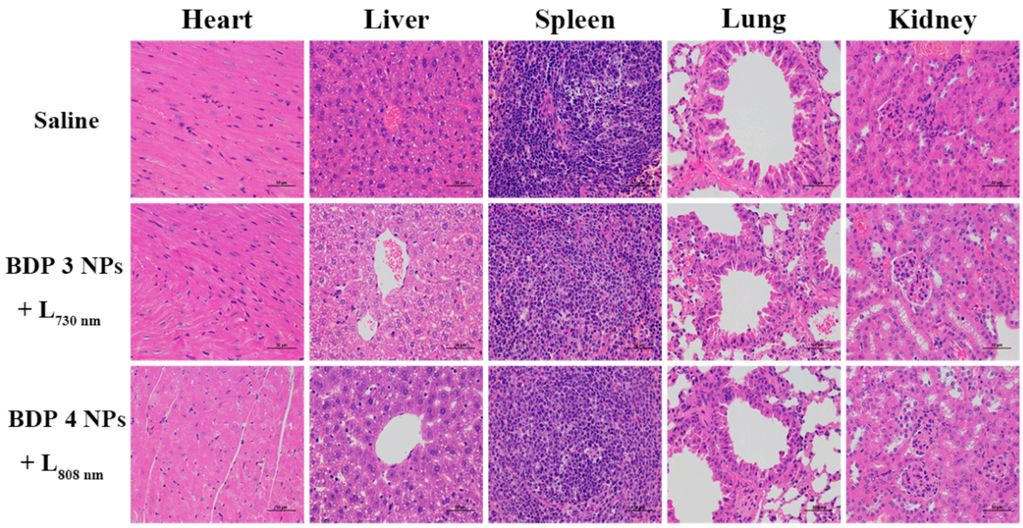

Figure S12. Biosafety evaluation: H&E staining of major organs after treatment under different conditions.

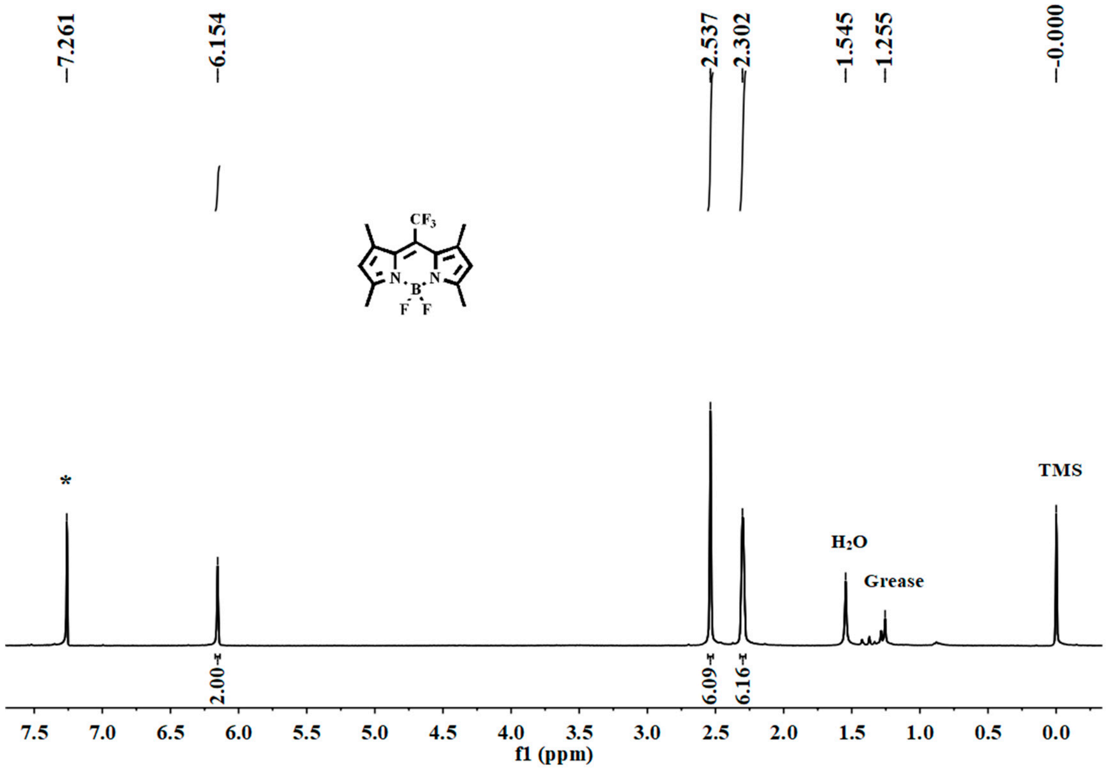

Figure S13. ^{1}H NMR spectrum of **BDP-CF$_3$** in $CDCl_3$.

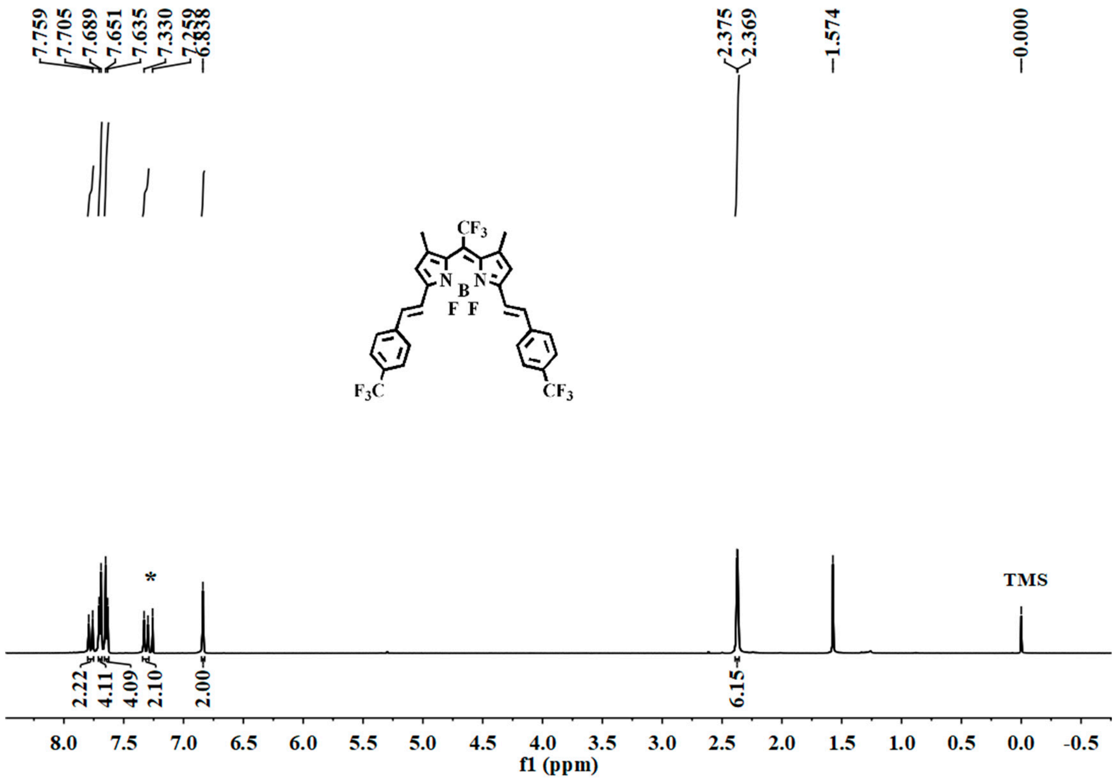

Figure S14. ^{1}H NMR spectrum of **BDP 1** in $CDCl_3$.

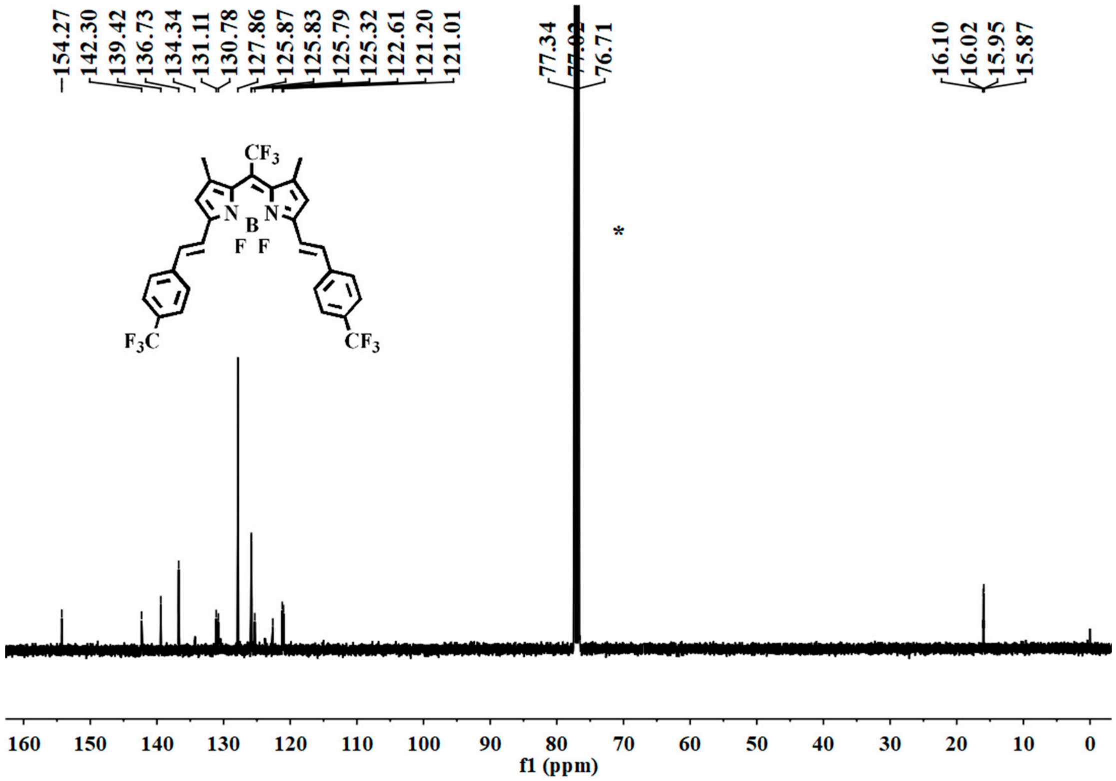

Figure S15. ^{13}C NMR spectrum of **BDP 1** in $CDCl_3$.

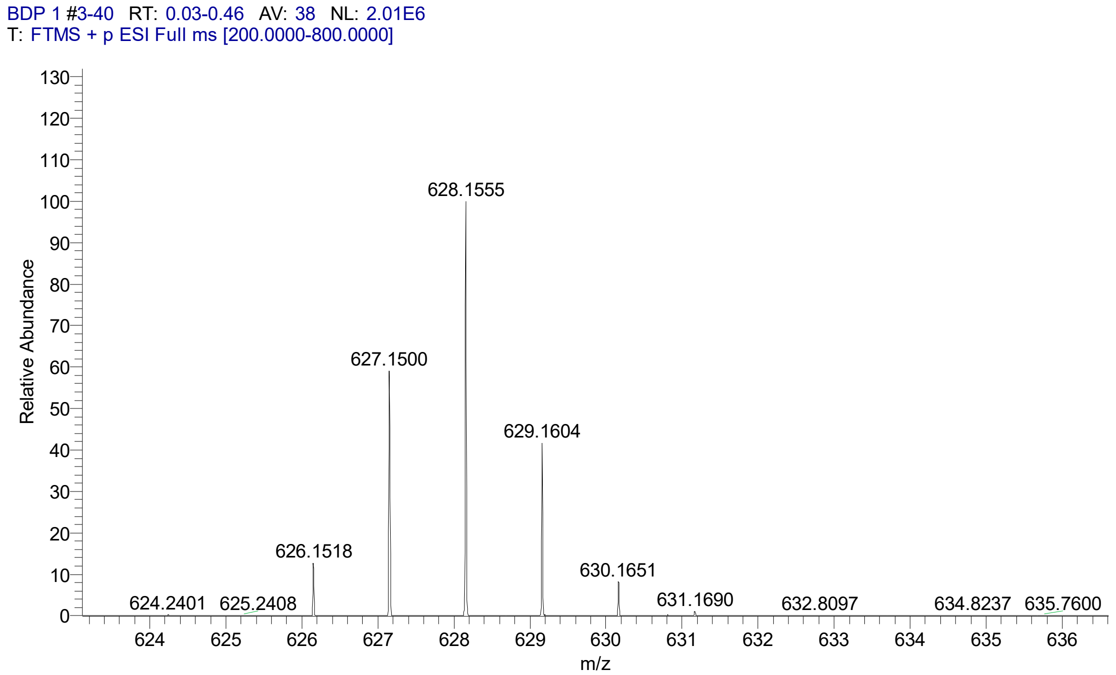

Figure S16. HRMS spectrum of **BDP 1**.

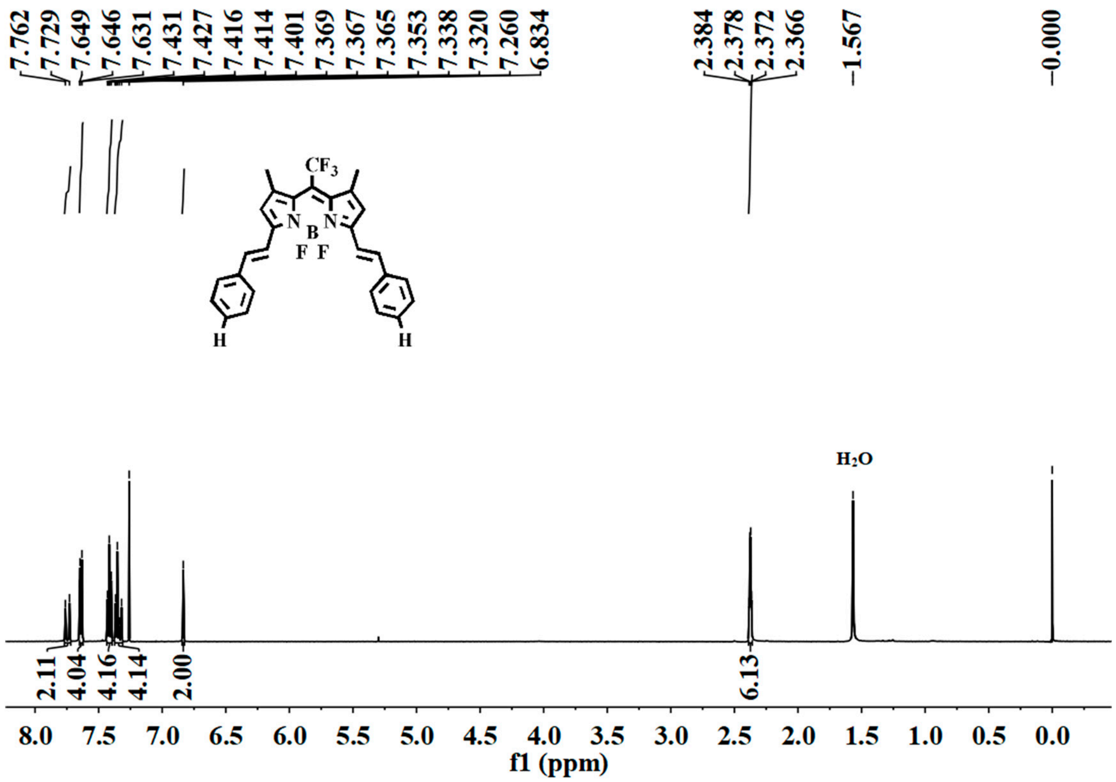

Figure S17. ^{1}H NMR spectrum of **BDP 2** in $CDCl_3$.

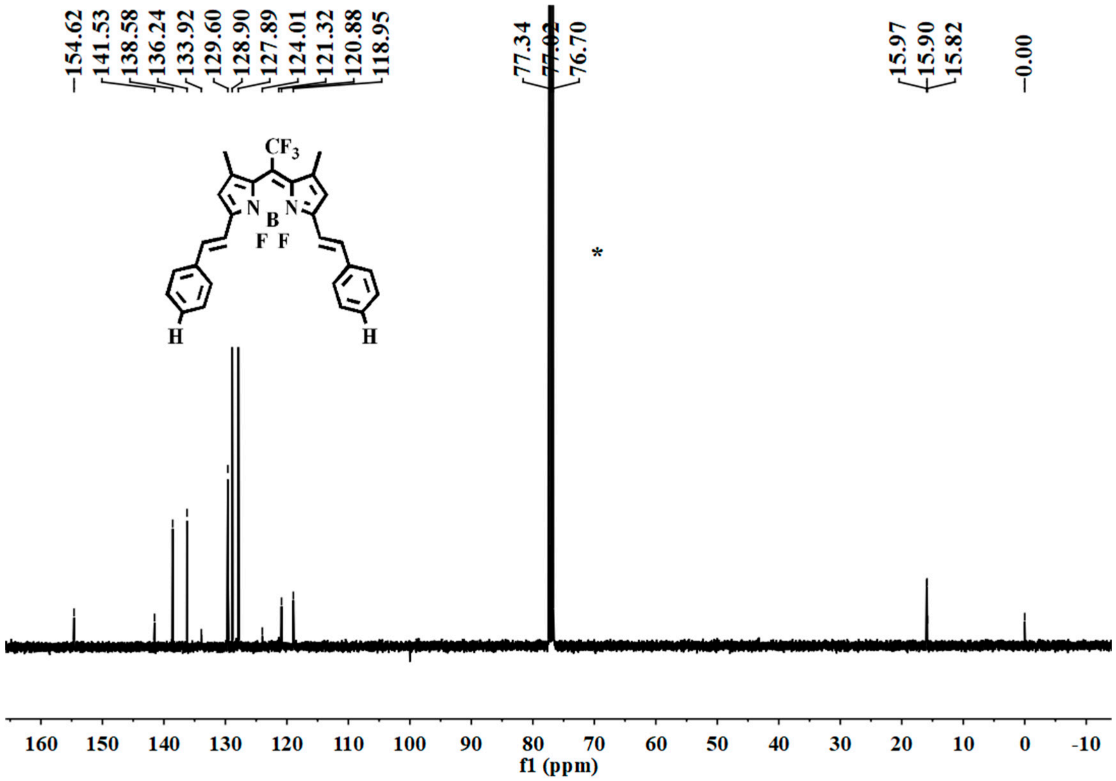

Figure S18. ^{13}C NMR spectrum of **BDP 2** in $CDCl_3$.

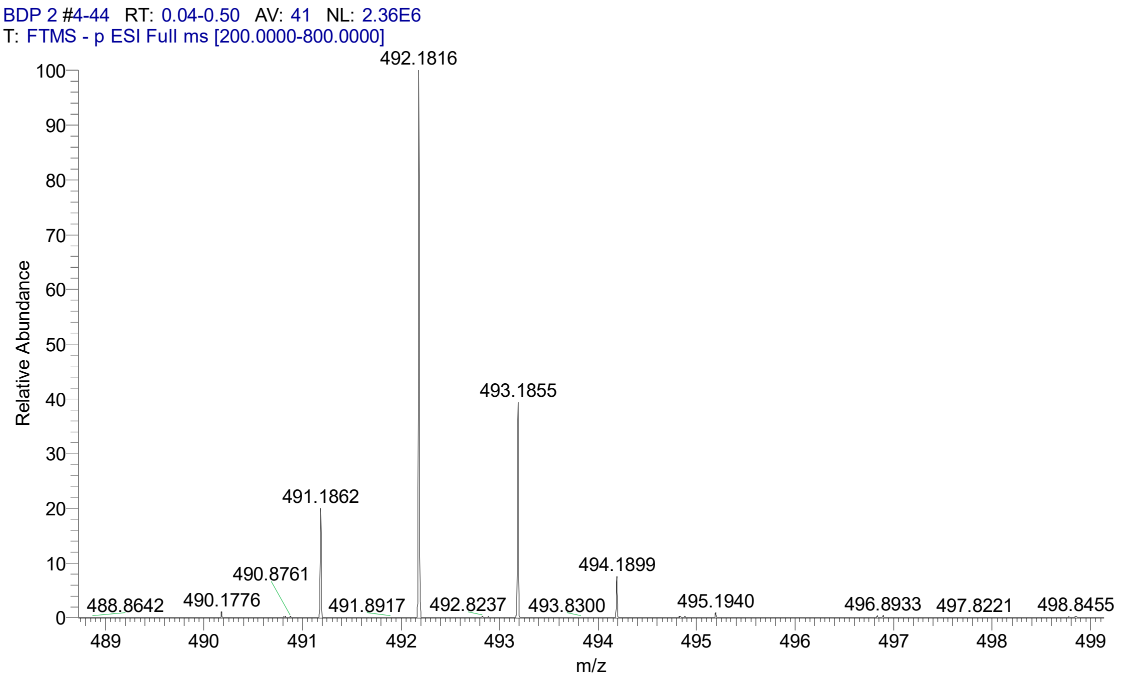

Figure S19. HRMS spectrum of **BDP 2**.

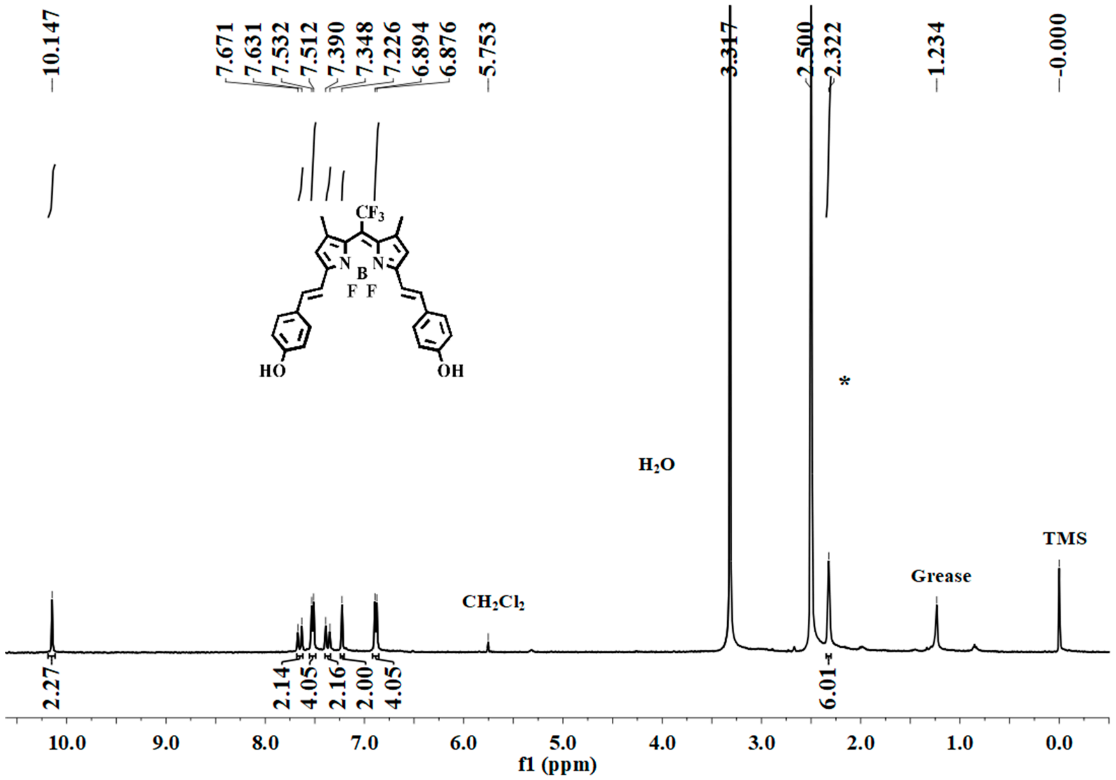

Figure S20. ^{1}H NMR spectrum of **BDP 3** in DMSO-d_6.

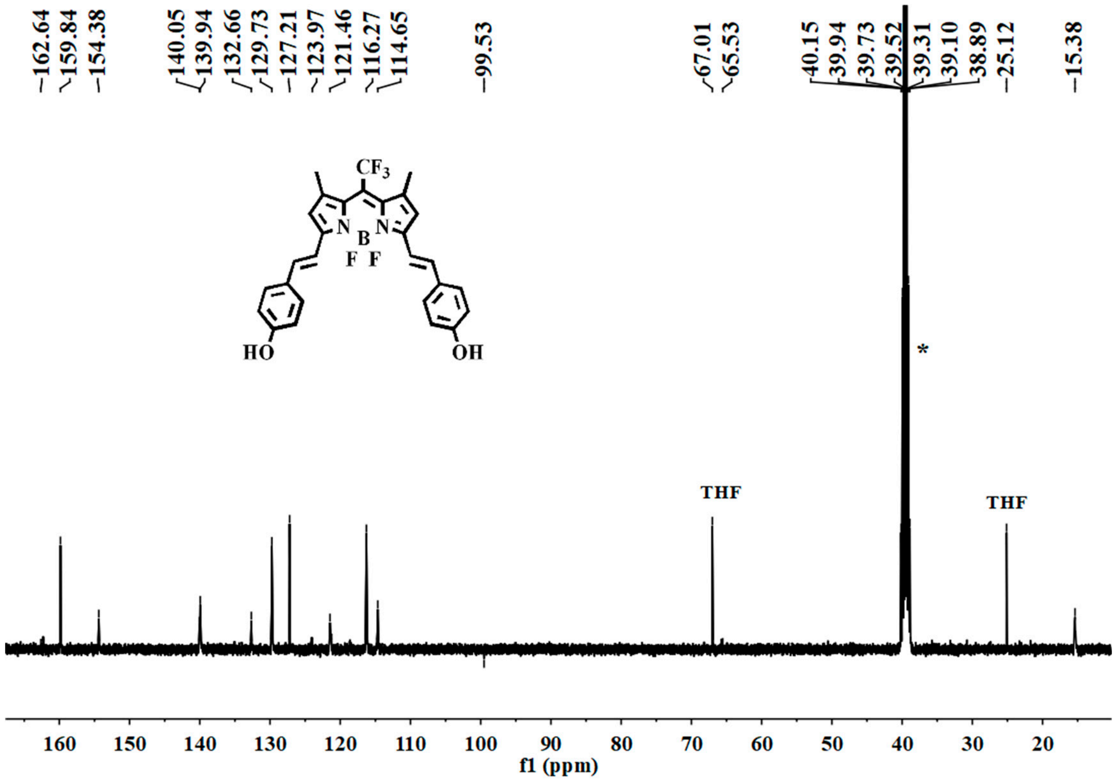

Figure S21. ^{13}C NMR spectrum of **BDP 3** in DMSO-d_6.

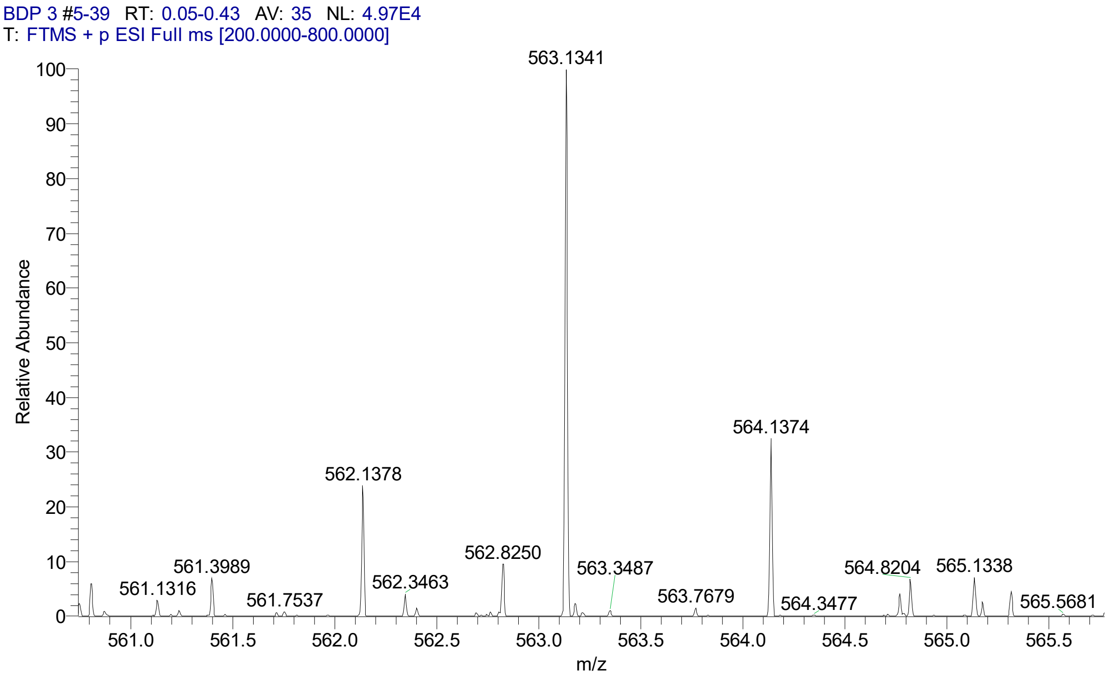

Figure S22. HRMS spectrum of **BDP 3**

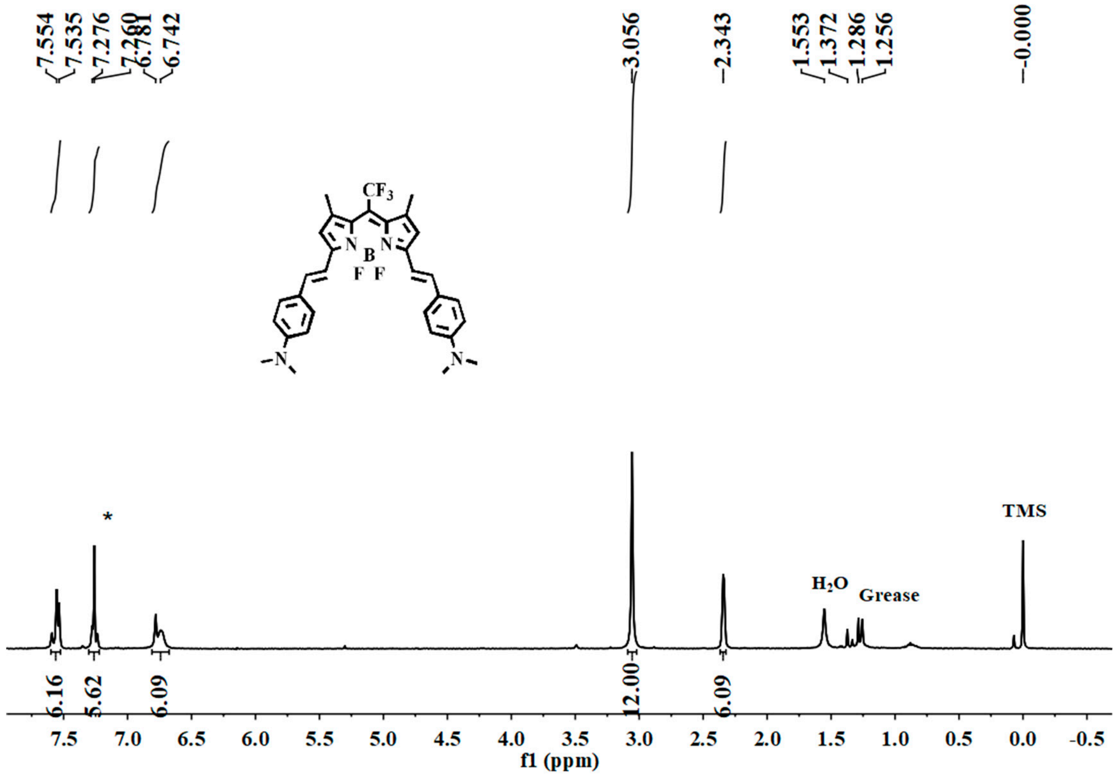

Figure S23. ^{1}H NMR spectrum of **BDP 4** in $CDCl_3$.
